# Human Exposures to Micro- and Nanoplastics in Water and Data Needed to Understand Potential Health Effects—A-State of the Science Review

**DOI:** 10.3390/microplastics4030060

**Published:** 2025-09-05

**Authors:** Max Zarate-Bermudez, Gaston Casillas, Janie Hils, Michael Yeh, Yulia Carroll

**Affiliations:** 1 National Center for Environmental Health, Centers for Disease Control and Prevention, Atlanta, GA 30329, USA; 2 Agency for Toxic Substances and Disease Registry, Centers for Disease Control and Prevention, Atlanta, GA 30329, USA; 3 Cincinnati Health Department, Cincinnati, OH 45229, USA

**Keywords:** micro- and nanoplastics in freshwater, micro- and nanoplastics in drinking water, micro- and nanoplastics in bottled water, exposure to micro- and nanoplastics, state-of-the-science review

## Abstract

Human exposure to micro- and nanoplastics (MNPs) in the environment and their potential health effects are of growing public interest. Regarding water, that interest grows because multiple studies found MNPs in different matrices including tap and bottled water. We intended to (i) understand how MNPs enter freshwater systems and drinkable water, (ii) assess the evidence of human exposure to MNPs in water, and (iii) identify data gaps to support the determination of potential health effects. We searched the literature and selected studies via rigorous inclusion criteria, analyzed the data assessing the reliability of findings, and identified data gaps associated with human exposure to MNPs in water. The lack of standard sampling and analytical methods for testing MNPs in water constitutes a barrier to make accurate comparisons. The diverse analytical methods to fully characterize MNPs led to different findings in samples of similar matrices. Current drinking and wastewater treatment systems are not designed to remove MNPs. However, efforts to enhance the precision and accuracy of MNPs’ characterization and their removal by treatment systems are promising. Therefore, addressing data gaps could produce reliable data for conducting exposure and risk assessments, protect our communities, and control the mobility of MNPs to minimize exposures.

## Graphical Abstract

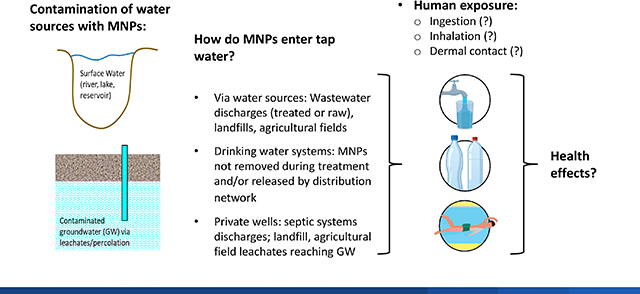

## 1. Introduction

Increased plastic production and consumption increased the generation and mismanagement of plastic waste. In the United States, approximately 36 million tons of plastic waste were generated in 2018 [1]. Also, primary and secondary micro- and nanoplastics (MNPs) are commonly found in the environment including freshwater systems, as is the focus of this review, raising public health concerns. Most authors indicate that MPs’ size ranges from 100 nm to ≤5 mm, while NPs’ size ranges from 1 nm to ≤100 nm. Primary MNPs, especially microbeads, which are used in various personal care products enter water sources mainly via wastewater discharges, solid waste disposal, and atmospheric deposition. Secondary MNPs are generated at a very slow rate through the breakdown of larger pieces of plastic waste via natural physical, chemical, and biological processes. Sunlight and heat can make plastics brittle and strong natural forces such as ocean waves, turbulent water streams, and winds can exercise enough mechanical stress to reduce them to MNPs.

Micro- and nanoplastics can reach freshwater systems through multiple pathways. They can drain from landfills and agricultural fields into groundwater through permeable soils and into nearby surface waters. Water streams are the main conduits for MNPs to reach estuaries, lakes, seas, and oceans while effluents of wastewater treatment plants (WWTPs) are considered the main source of MNPs entering natural waters. Even with removals of 97% through tertiary treatment, effluents could still release billions of microplastics (MPs) into receiving waters [2].

Therefore, our objectives focused on systematically review the literature to assess if the available data provide evidence to determine the following:

- The potential routes of human exposure to MNPs in freshwater and the potential health effects including laboratory-scale studies,
- The pathways MNPs follow to reach freshwater systems,
- The pathways MNPs follow to reach drinking water for the design of preventive measures, and
- The data, which are needed to better understand human exposure and potential health effects.

Guided by our objectives we assessed the validity of study findings on levels of MNPs in drinking water (tap and bottled), drinking water treatment plants (DWTPs), and sources of MNPs entering freshwater systems like WWTPs’ effluents. Also, we assessed the validity of methods for sampling and sample preparation as well as for quantifying and the physical and chemical characterization (full characterization) of MNPs.

In this review, we aimed for studies showing direct evidence of human exposure to MNPs in water during 1990–2022. We focused on detailed descriptions of the methods for sample collection and preparation and for the quantification and full characterization of MNPs in water of different matrices. This targeted approach also aimed to identify gaps that need to be addressed to inform the understanding of human exposure to MNPs in water and their potential health effects.

## 2. Methodology

### Literature search

We published and registered our systematic review protocol in PROSPERO (ID: CRD42021278806). For the literature search, we used Medline, Embase, CAB Abstract, CINAHL, GreenFile, Environmental Science Collection, Scopus, Compendex Engineering Village, and Cochrane Library. A total of 6472 scientific papers met our search criteria (Table S1) for the period 1990–2022. They focused on drinking water, drinking and wastewater treatment systems at both real and laboratory scales, and freshwater systems. Papers were saved in an EndNote library that is compatible with Covidence, the electronic platform that we used to conduct this review. Also, Covidence is compliant with PRISMA 2020 guidelines (see PRISMA 2020 check list in Supporting Documents 2).

### Study selection for data extraction

We uploaded the EndNote library into Covidence, and 55 duplicates were identified and eliminated. Two reviewers made independent determinations per paper at each stage of the review. In case of a disagreement, a third reviewer made the final determination. We screened 6417 titles and abstracts (stage 1) and reviewed 744 full texts (stage 2). Our exclusion criteria focused on literature reviews and papers that (i) did not focus on freshwater systems or drinking water and water-based beverages; (ii) lacked a methods section or methods section lacked details useful for replication; (iii) expressed concentrations of MNPs in units other than mass or number per volume of water; (iv) focused on MNPs removal without reporting concentrations in raw and/or treated water; (v) did not address the study objectives in the findings and/or discussion sections; (vi) did not inform our understanding of human exposures to MNPs; and (vii) were not in English, Spanish, or Portuguese. We selected 253 papers upon full text review and developed templates for quality assessment and data extraction (stage 3).

### Quality assessment

**Figure 1.**
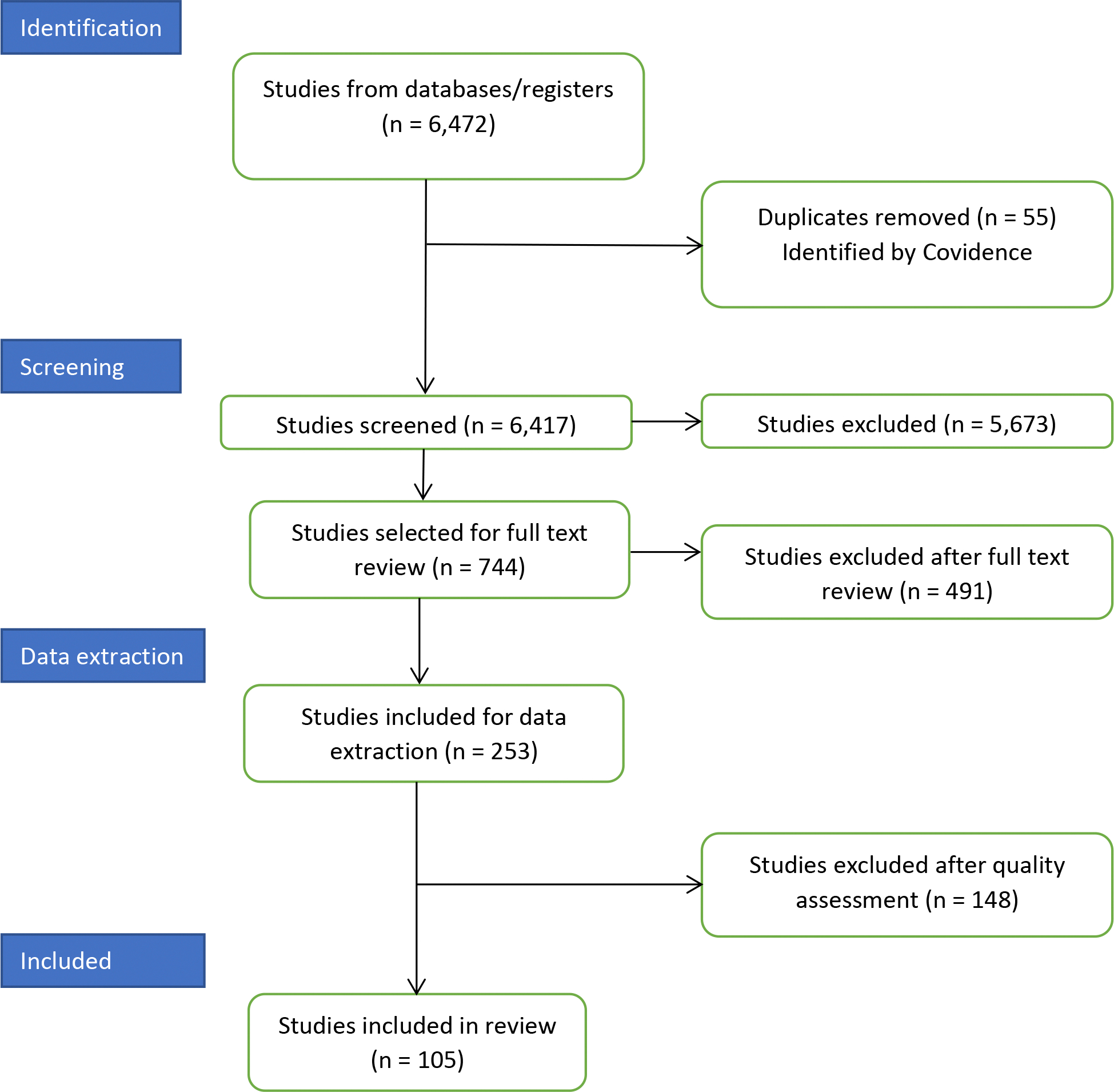
Covidence’s PRISMA flowchart for the selection of papers to be included in the review

The absence of standard methods for sampling and characterizing MNPs made us focus our quality assessment of the papers on the (i) detailed description of both sampling and analytical methods to fully characterize MNPs, (ii) connection between objectives, findings, and discussion sections, and (iii) balanced selection of the literature to enrich the discussion and inform our knowledge on MNPs in water. We excluded 148 papers by quality assessment and selected 105 papers published during 2016–2022 (Figure 1).

### Data extraction and summary

**Figure 2.**
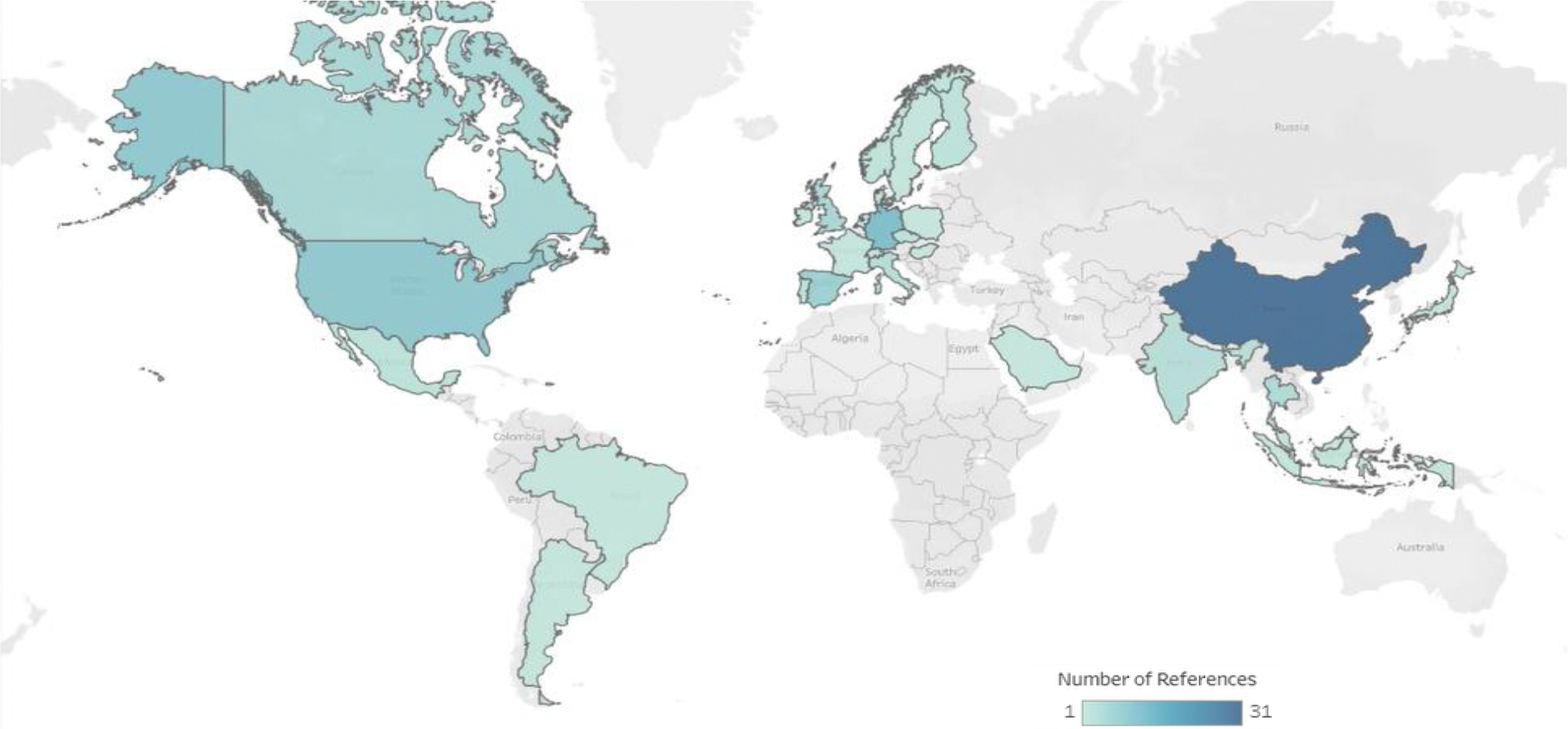
Map summarizing location of scientific publications selected by country

To assess the current knowledge on pathways of human exposure to MNPs in drinking water and freshwater systems, and determine the potential human health effects, we summarized data from the 105 papers selected. We extracted and assessed data on MNP concentrations in (i) drinking water from taps, bottles, and water-based beverages; (ii) freshwater systems; and (iii) effluents from WWTPs. Also, data from laboratory studies focused on (iv) toxic effects observed in human cells when exposed to MNPs; (v) health effects potentially linked to plastic additives that leached into water; and (vi) advances in sampling and analytical methods. Finally, data on (vii) the removal of MNPs during drinking and wastewater treatment at real and laboratory scales. Figure 2 shows the geographic location of the studies selected for this review. Additionally, we summarized data from six papers that were published recently and were not part of our initial search. However, they have been (i) recognized as influential in shaping research on MNPs in water, or (ii) informative to our understanding of human exposures to environmental MNPs.

## 3. Findings and Discussion

We found that authors used a variety of sampling methods across different water matrices and among waters of similar matrix. Also, we found that the methods used for the full characterization of MNPs in water vary widely. Variations occurred in sample preparation, isolation of suspected MNPs for physical characterization, and their selection for polymer identification. Authors used various microscopic methods to count and measure the length of MNPs and most used scanning electron microscopy (SEM) to count and measure the surface topography of MNPs. The spectroscopic methods mostly used for polymer identification included Fourier Transform Infrared (FTIR) spectroscopy, FTIR microscopy or μ-FTIR, attenuated total reflectance (ATR)-FTIR, Raman spectroscopy, Raman microscopy or μ-Raman, and energy dispersive X-ray spectroscopy (EDS). The polymers and their acronyms reported are listed in Box 1.

Micro- and nanoplastics are pervasive in tap and bottled water and in freshwaters across the world at varying levels. We did not find papers focused on direct human exposures to either MNPs or plastic additives via ingestion, inhalation, or dermal contact. However, the focus of 10 selected papers was on potential health effects in controlled laboratory studies.

### MNPs in drinking water systems

We selected 19 studies focused on the full characterization of MPs in samples of drinking water systems. Authors characterized MPs in samples from taps or the entire system and some estimated MPs removal efficiencies by DWTPs. Authors used microscopic methods to count and physically characterize (size, usually the length or diameter, and shape) MPs; counts and physical characteristics varied widely. To characterize MPs surface morphology, authors used SEM or combined SEM, microscopy, and energy-dispersive X-ray analysis (EDX or EDS). Authors used spectroscopic methods or pyrolysis-gas chromatography-mass spectrometry (py-GCMS) to characterize MNPs chemically or identify polymers. Discriminating true from suspected MPs is very important for exposure and risk assessments but was less frequently reported. Characterizing MPs in drinking water requires thorough planning and reporting to produce reliable estimates of human exposure to MPs and to assess potential health effects.

Sampling and sample preparation methods are not standardized as shown in Table 1. The number of sampling sites and samples, sample volumes, and collection methods varied widely. Sample preparation, especially digestion processes also varied but wet peroxide oxidation dominated. Tests were conducted in single, duplicate, or triplicate samples. Bottles for sample collection were of plastic, glass, or aluminum even during the conduct of one single study. Also, the size range of MPs was wide, 6.5 μm to 5 mm.

**Figure 3.**
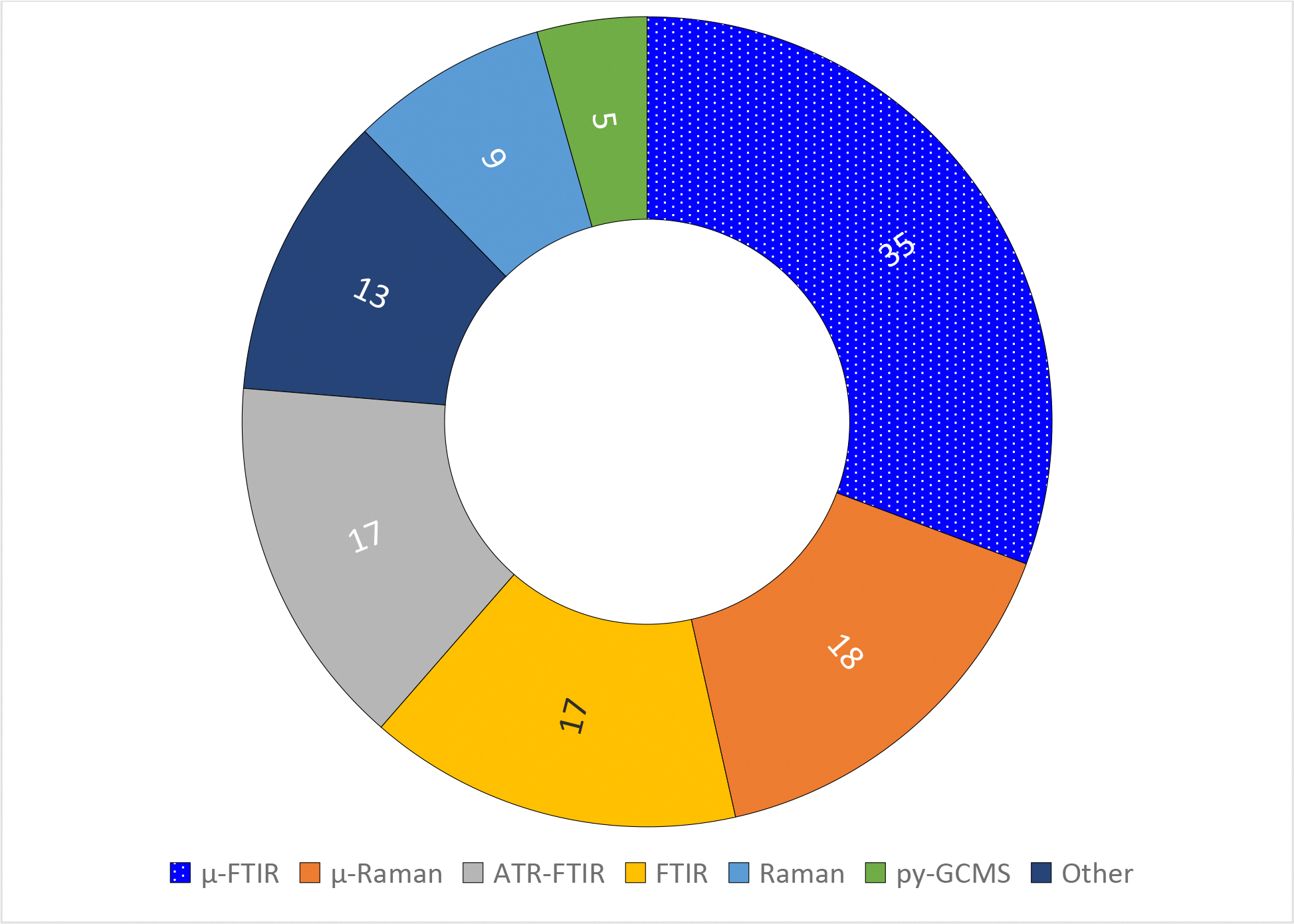
Methods used to identify polymers in the selected papers (n = 114; 17 out of 98 authors reported using two identification methods)

Figure 3 shows that methods used to identify polymers varied widely. To date, there are no standard methods for polymer identification in MNPs. Spectroscopy remains dominant, particularly the FTIR- and Raman-based methods for polymer identification. Some authors who used FTIR-based spectroscopy reported spectral matches that ranged ≥60% to ≥75%. Also, those who estimated percentages of true MPs found variations from 46% to 65% for tap water. Spectroscopy and py-GCMS are analytical methods used to identify polymers, thus findings could help estimating percentages of true MNPs in water samples. Polymer identification and density, dimensions to estimate MNP’s volume, percent of true polymers, and sample volume are needed to convert expressions of number of MNPs per volume into mass per volume units.

### 3.1. MNPs Identification in Samples of Treated Water, House Connections, and Tap Water

Authors who tested for MPs in samples of treated water, house connections, or tap water identified polymers using spectrometry. Some used FTIR-based methods to identify PE, PAM, and PVC [3], PE only [6], or PP, PS, and PET [7], and reported the percent of the suspected microfibers and MPs (MFPs) tested; mixed MFs and MPs (MFPs), are commonly found in water samples of all matrices. These authors also demonstrated that visual analyses using only microscopy could lead to false positives of MNPs in water.

The use of focal plane array (FPA)-μ-FTIR to quantify and fully characterize MPs assessing the influence of the age of distribution systems was studied in a high performance DWTP [14]. Counts of MPs in the older distribution system were >40 times greater compared to the newer system. Using py-GCMS, they identified PEST, PA, PMMA, PVC, PS, and PE. They found that the mass of MPs in distribution systems was 20 times greater than the mass in outlets stating that the age of distribution systems may influence the release of MPs. Others used μ-Raman and identified PTT [4], PE, PP, and PE-PP blend [5] in treated water, but some reported no MPs after blanks correction [8].

The likely sources of MFPs were found to be abrasion of pipes and distribution system fittings, and the use of enhanced flocculation chemicals (PAM). Authors who implemented quality assurance/quality control (QA/QC) protocols, used acceptable measures, which varied widely to support their findings. A rigorous QA/QC protocol enhances the reliability of findings when conducting analytical work. Thus, accurate assessments of human exposure to MNPs in water and their potential impact on human health require standard methods for sampling and analyses.

### 3.2. MNPs, from Water Sources to Tap

Table 1 shows the variation in sample volumes collected from water sources, DWTPs outlets (treated water), and taps to test for MPs. Authors used μ-FTIR, μ-Raman, and a combination of both to identify polymers. They used μ-Raman for MPs < 300 μm, μ-FTIR for MPs 25–500 μm, or combined microscopy with ATR-FTIR for MPs ≥ 300 μm.

Authors use μ-Raman to test for MPS < 20 μm, μ-FTIR for MPs ≥ 20 to 500 μm, and ATR-FTIR for MPs ≥ 500 μm to 5 mm in water samples. Spectral matches for polymer identification have been reported at ranges > 70%, considered acceptable for MNPs. Only a few authors used py-GCMS to identify polymers and determine the mass of MNPs.

Use of μ-Raman and μ-FTIR remain the preferred methods to test for and fully characterize MPs in drinking water systems. Some authors reported using FPA-μ-FTIR to identify PEST, PA, and PVC in treated water [10]. Among authors who used μ-FTIR, some identified rayon, PET, and PE in tap water and estimated an overall MPs’ removal of 99.8% [12]. Others identified PE, PET, PP, and PA in treated water and estimated an overall MPs’ removal of 87.4% by the DWTP [15]; they found PVC in the tap water and attributed it to the PVC pipes of the distribution network. Authors also identified PEST, PS, and nylon in tap water, PEST, PP, PVC, and nylon in treated water, and PVC, PE, PP, and nylon in pipe scales of the distribution system. The estimated MPs’ removal of <30% by the DWTP’s grew to ~90% due to deposition of MPs onto the pipe scales [16]. Thus, understanding the mechanisms by which MPs attach onto pipe scales may be helpful in enhancing the removal of MPs by DWTPs. In addition, conducting a survey of the drinking water system and planning the sampling and analytical work can lead to reliable results.

Removals of MPs ranged 74% to 86% in treated water of three DWTPs where PET, PP and PE were identified using μ-Raman [9]. Earlier they identified cellulose acetate, PE, PET, and PVC in treated water of two DWTPs with removal efficiencies of ~40% and ~90% [11]. Others studied the seasonal influence on MPs levels in canals and DWTPs. They estimated poor removals of 27.7% and 12.7% during the dry and wet seasons, respectively, and identified PE, PET, PP, PS, and PVC in tap water [13]. Those findings confirm that conventional DWTPs were not designed to remove MNPs, which deserves upmost attention from the drinking water treatment industry.

### 3.3. DWTPs Efficiency in Removing MPs

Findings of five studies focused on DWTPs’ efficiency to remove MPs are shown in Table 1. Methods to fully characterize MPs, and number of samples and volumes varied widely. Some authors implemented QA/QC protocols to enhance the reliability of results.

Authors who used a FTIR-based spectroscopic method and implemented a QA/QC protocol produced reliable results. Some achieved efficiencies that averaged >92% to remove ABS and PS by eight DWTPs [17] and PES and PP by an advanced and by an upgraded conventional DWTP [18]. Lower efficiencies for cellophane removal by conventional DWTPs were achieved, 54% for DWTP 1 and 76% for DWTP 2 [21]. As above, these findings reveal the need for enhancing the removal efficiency of MPs by DWTPs.

Learning about seasonal influences on the fate of MPs in water expanded to assessing DWTPs’ removal efficiencies. Removals of 67.6% were estimated during the dry season and of 57.2% during the rainy season; using ATR-FTIR and confocal Raman spectroscopy authors identified PE, PP, PVC, PET, and PS in treated water [20]. Others focused on enhancing the reliability of results by implementing QA/QC protocols. Using py-GCMS, authors estimated removal efficiencies that varied from 43% to 100% and identified PE and PA in treated water of a DWTP [19].

### 3.4. Efficiency of DWTPs in Removing MNPs at Laboratory-Scale

Findings from laboratory-scale studies on MNP removals simulating DWTPs processes are shown in Table 2. Some studied how magnesium/aluminum (Mg/Al)-layered double hydroxide (LDH) formation can remove >90% PS NPs due to electrostatic adsorption and intermolecular forces [22]. Others assessed dosages of PAM, sodium alginate, and activated silicic acid (ASA) affect coagulation by poly-aluminum chloride to remove PET MPs. At dosage > 50 mg/L, PAM achieved the best removal, 91.45% [23]. Removals of ~86% for PE, PP, and PS and of ~63% for PET were achieved using magnetic nano-iron oxide (Fe_3_O_4_) by magnetizing MPs [24]. Also, the effects of ozonation or chlorination on PS NPs were tested; ozonation degraded the NPs molecular weight (MW) and mineralized them 12.5 and 10 times greater, respectively, than chlorination [25]. The processes studied could be applied at real scale. As mentioned above, conventional and advanced DWTPs could benefit from them since they are not designed to remove MNPs from the raw water.

### MNPs in drinking water taps, bottled water, and water-based beverages

We summarized data from 13 studies that focused on detecting MNPs, 1 nm to 5 mm, in bottled water and water-based beverages. Again, we found a wide variation in sampling methods, number of samples and sample volumes collected, and analytical protocols (Table 3). The issue of MNPs in bottled water and water-based beverages is universal.

The occurrence and nature of MPs in tap water, bottled water, and beer from dozens of countries were tested [26]. Authors could not identify the polymers but implemented a QA/QC protocol to prevent cross-contamination, count, and physically characterize MPs. Prior to 2020, most authors used a FTIR-based method to identify polymers and estimate the percent of true MPs. Some found that <50% of MPs > 100 μm were true MPs, mainly PP. When including MPs < 100 μm, that percent was expected to increase but polymer identification was not possible by FTIR-based methods [27]. They also found larger levels of MPs in water of the same brand but bottled in plastic rather than glass, suggesting that plastic bottles can release MPs into the water.

Levels of PET and PEST MPs in water of returnable plastic bottles were 2.4 and 8.4 times greater than in water of glass and single-use bottles, respectively [28]. Authors suggested that carbon dioxide may play a role in releasing MPs from plastic bottles because the levels found in sparkling water were ~8 times greater the MPs level in still water. They also found PE, PEST, and PP MPs in carton beverages. Thus, the presence of MPs in bottled water and water-based beverages was demonstrated as early as 2018 and the focus on fully characterizing MNPs continued making progress.

Authors identified different polymers in water of bottles of different materials, PET, PA, PS, PE, PU, and PP in PET bottles, PP and PET in glass bottles, and PE and PS in PC bottles [6]. All bottles had HDPE caps, which revealed that identifying the MPs’ source is important. Others described sampling methods, sample preparation, and analytical procedures in detail and pointed at the filling and/or the capping processes of four water bottlers as critical entry points of MPs into the water [29].

Some authors combined spectroscopic methods. Using ATR-FTIR for MPs ≥ 50 μm and confocal Raman spectroscopy for MPs 1–50 μm led authors to find that <50% of the suspected MPs were true polymers [30]. They identified PET, PE, PP, PA, PVC by ATR-FTIR but did not report the Raman spectroscopy results. Thus, the need for reporting levels of true polymers in water is crucial for planning human exposure and risk assessments.

Authors who pioneered the characterization of MPs ≥ 1 μm in bottled water using μ-Raman identified PET in water of PET bottles and PE and PP in water of glass bottles [31]. Levels of MPs in water of glass bottles were greater than those of PET bottles. Others identified PA and PEA in beer and energy drinks. Beer had greater levels of MPs than energy drinks [32]. Also, a detailed sampling and analytical protocol allowed authors to find PVPP, a membrane filter stabilizer, in the beer [33]. It revealed that filters can leach additives into beer or other water-based beverages.

The release of MPs during formula preparation from baby feeding bottles (BFBs) was also studied [34,35]. Li et al. [34] achieved >92% recoveries and found ≤10^7^ MPs/L after mimicking formula preparation; spectra seemed to match PP and PE. Song et al. [35] found that opening and closing bottles 100 times can release ≤10^5^ MPs/L from BFBs and ≤10^4^ MPs/L from PC and PP water bottles. They identified PVC, PA, PU, PET, PS, and PE using laser direct infrared (LDIR)-μ-FTIR and found that <45% of the suspected MPs were true polymers. Estimated monthly ingestions were 117.3 and 16.3 MPs (<100 μm) for infants and children, respectively, via BFBs and water bottles.

Also, the release of MPs when applying mechanical stress to single-use mineral water bottles and the presence of lubricants in HDPE caps were studied [36]. Using SEM and EDS they determined that opening and closing bottles 100 times released >10^6^ PET MPs < 5 μm; they used GC-MS to find that caps released the lubricant behenamide. And the release of MNPs from PET and nylon teabags free of tea leaves when brewing one cup of tea was also studied [37]. They found millions of MPs < 150 μm and billions of NPs < 1 μm, estimated to be 16 μg of MNPs, were released by one teabag. The polymers released matched the teabag composition by ATR-FTIR and X-ray photoelectron spectroscopy (XPS). Thus, fully characterizing MNPs in drinking water or water-based beverages, reporting their levels as mass or number per volume, the percent of true polymers, and polymer density are needed to make conversions useful to plan exposure and risk assessment studies.

### MPs in sources of drinking water

#### Rivers and other water streams

We summarized data from 28 studies covering the full characterization of MPs in rivers. Some authors characterized MPs in rivers, from source-to-sink, or in canals and streams influenced by stormwater runoff. Others characterized MPs of anthropogenic origin to understand the influence of urban settings on the levels of MPs in rivers or urban canals. The influence of WWTP discharges seasonal weather events on MP levels in receiving waters was also studied. The size of MPs varied from 10 μm to 5 mm and polymers were identified mostly by spectroscopic methods and some by py-GCMS. Variations from sampling to analytical methods, MP levels found, polymers identified, and percent of true polymers are described below and shown in Table S2.

Some authors used ATR-FTIR to identify polymers reporting that percentages of true polymers in suspected MPs differ greatly. It varied from 7%, identifying PE, PP, and PS [38] to 79%, identifying the same polymers plus PET [39]. Others studied the effects of rainy season on the quantity and quality of MFPs and found that pre-monsoon MFPs’ levels almost doubled the post-monsoon levels [40]. They identified rayon, PMMA, PET, PVC, PEST, and nylon.

Authors who used μ-Raman to quantify MPs and identify polymers reported findings that varied widely. Some identified PP, PE, PE-PP blend, PESU, PS, and PET [41]. Others indicated that sample type and preparation influenced findings and could not detect MFPs in undigested samples [42]. However, in digested samples PA and PET were dominant polymers in MFs and MPs, respectively.

The effect of sampling methods on MFPs distribution was studied in pump- and plankton net-collected samples using μ-FTIR [43]. Levels of MPs in pump-collected samples were 100 times greater than levels in plankton net-collected samples (nets of 75 μm and 300 μm mesh). The polymers identified were PP-PE blend and PE and 98.6% of the suspected MFPs were true polymers.

### 3.5. MNPs from Water Sources-to-Sink

Learning about the fate of MNPs on their way to the oceans is important for assessing potential human exposure in recreational waters and their uptake by aquatic animals. Some identified PET and PTFE in MFs of a river and found that 50% of the suspected MFs were true polymers using μ-FTIR [44]. They estimated an average daily load of 3 × 10^8^ MFs to the Atlantic Ocean. Others identified PP, PE, PET, and PEST by μ-Raman in grab and net-collected samples from rivers flowing to the ocean [45]. Grab samples had greater MP levels than net samples, which was similar to MP levels in grab samples compared to trawling samples [46]. In the latter case, authors used FTIR-based spectroscopy to identify PP, PE, PS, PE/PP, and PVC along a river. The findings above confirm the importance of planning the sampling and testing when designing studies to characterize MNPs in water.

Authors also characterized MPs in streams nearby agricultural fields by combining μ-Raman and ATR-FTIR; they identified PVC, PP, PMMA, PE, PE wax, PTFE, PU, and PA in a small subsample [47]. Others confirmed the influence of human activities and population size on the levels and types of MNPs found in nearby rivers. Using FTIR-based spectroscopy, some identified PE and PET suspecting clothes washing and solid waste mismanagement as the main sources [48]. Others identified POE, PET, PUA, EP, PF, PA and PE MPs with the highest levels in samples near urban areas and tributaries confluence [49].

### 3.6. MPs of Anthropogenic Origin and the Influence of Urban Settings on MP Levels

Some authors used FTIR-based spectroscopy to characterize MFPs in rivers and identify factors of anthropogenic origin that may influence their levels. The dominance of MFs, not MPs was observed in urban canals but the polymers were not identified [50]. Others assessed the annual flux and seasonal and sectional distributions of MFs in a river and identified PET, PP, PA, and PET-PUR blend [51]. Also, PES MFs and PE, PP, and PE/PP blend MPs were identified in samples from canals and a river near a populous city [52]. Most of the MFPs (>91%) were synthetic and MFs were dominant. Laundry and WWTPs effluents were the suspect sources.

The selection of sampling points along urban rivers can be an important predictor of MFPs levels. High MFP levels in samples downstream from the confluence of tributaries and from discharges of WWTPs’ effluents were reported [53]. About 72% of the suspected MFPs were true polymers identified as PP, PE, and PET. The influence of sampling methods was also studied. Levels of MFPs in pump-collected samples were again much greater (245 times) than levels in samples collected using manta trawl of 333 μm mesh [54]. The authors pointed at discharges of WWTPs as the most likely source and PE, PE-PP blend, PA, PP, PS, PU, and PET MPs were identified. In samples collected by an ichthyoplankton net (250 μm), PP and PE of low, medium, and high densities were identified [55]. Levels of MPs in upstream samples, near a combined sewage overflow doubled the levels in downstream samples. Others found that samples collected near industrial waste discharges had the highest MPs levels, identified PP, PE, PS and PVC, and found that 88.6% of the suspected MPs were true polymers [56]. Those findings reveal the inverse relationship between nets’ sieve size and the MPs levels in water samples.

The microbeads levels in a river were determined by μ-Raman or ATR-FTIR identifying PS or PS-divinylbenzene copolymer in 95% of them [57]. Samples downstream from a WWTP discharge had the highest microbeads levels, so WWTP and industrial discharges together with industrial runoff were the most likely sources. The MFPs in samples from protected zones of a river were characterized and identified using Raman spectroscopy [58]. The highest levels, near the confluence of tributaries, helped in developing a model to predict MFPs’ peak fluctuations. Also, PE, PET, PP, and nylon were identified.

Authors used μ-Raman to characterize MFPs in samples of a river along urban areas and implemented a QA/QC protocol, which included spike recovery, to validate their test results [59]. They identified PES, cellulose acetate, and PP in MFs and PE, PP, and PE/PP blend in MPs. Also, the temporal and spatial influence of tributaries on the levels of MFPs in rivers was studied [60]. Flood events influenced the levels of MFPs and polymer identification was performed using FTIR. They found that 96% of the suspected MFPs were true polymers. Others observed seasonal, population density, and land use influences on the MFs levels in rivers [61]. They used Raman spectroscopy to identify PS, PP, PE, and nylon and used μ-FTIR to verify the nylon MFs.

### 3.7. Other Factors That May Impact the Levels of MNPs in Rivers

The influence of WWTPs’ discharges on MFP levels in rivers and other streams was demonstrated [62,63]. McCormick et al. reported that MFP levels upstream from WWTPs discharges were almost half the levels downstream. They reported that MPs can be vectors of microbial pathogens, the dominant species growing on MPs. Also, using py-GCMS they identified PE, PP, and PS. Schell et al. used μ-FTIR to characterize MFPs and determine their spatial and seasonal distribution. Samples downstream from WWTPs discharges had the highest MFPs level and PE, PP, PES, PMMA, and PS were found in WWTPs effluents.

The influence of precipitation on levels of MFPs in rivers was studied [64,65]. Cheung et al. assessed the impact of heavy rainfall on MP levels of a river by counting MPs of size 0.71 to 4.75 mm through the naked eye and MPs < 0.710 mm using microscopy. They found that MP peaks could occur during and shortly after heavy rain by observing 10-fold level decreases within two hours of the rain. Using ATR-FTIR, they identified PP/EPR blend, most likely from adjacent roads and reported that 76.1% of the suspected MPs were true polymers. Wang et al. tested for MFPs in the top surface of a river during dry and wet seasons in samples from upstream, midstream, and downstream sites. They found slight differences in MFP levels and polymer composition between the dry and wet seasons. Using μ-FTIR, authors identified PP, PE, PS, PET, PVC, and PA (dry season) and PP, PET, PS, PE, PA, and PVC (wet season).

### Lakes, ponds, and reservoirs

Topics of study on lakes, reservoirs, and retention ponds ranged from quantifying the MFPs to determining their sources and contributors, physically and chemically characterizing them, and assessing the atmospheric influences transporting MFPs (Table S3). Again, we noted that sampling and analytical methods also varied widely identifying the need for standardizing those methods for different water matrices.

The influence of influent water from rivers, smaller streams, and WWTPs’ discharges on MFP levels of lakes was largely studied but a few authors provided a detailed methodology to support their findings. Authors used μ-FTIR to fully characterize MFPs, some found the highest MPs’ levels in inflow samples and identified PE, PS and PET [66]. As discussed above, sampling methods were also compared finding that samples collected by the pump-filtration (300, 100, 20 μm) method had ~600 times greater MFP levels than the manta trawl (333 μm) samples [67]. They identified PET, PAN, PP in MFs, and PP, PE, PMMA, PVC, PS and PET in MPs and WWTPs discharges were the likely sources. Others characterized MFPs in lakes of remote areas to assess the atmospheric influence on their transport. Similar MFP levels were found in a lake’s main hole and in the outlet [68]. Very low true polymers were found, 18%, mainly PE and PP, and 11% of the suspected MFs, mainly PP and PS. Others reported finding MFs in a lake and identified numerous polymers, PET, PP, nylon, PAN, UF, PEP/PEPD, PVC and PE [69].

Authors also combined μ-FTIR and μ-Raman to fully characterize MPs of different size in lakes and found the highest levels of MPs near densely populated areas or the WWTP effluent discharge into a lake. Some pointed at influent rivers and WWTPs effluent discharges as MP sources [47,67], identified cellulose, PET, PE, PVC, and PP in suspected MPs and reported the detection of PVC additives and dyes [67]. Others identified PP and PE and reported that 74% of the suspected MPs were true polymers [70].

Scientists studied MPs in Chinese reservoirs. The highest levels were near urban areas [71] and near densely populated urban areas and a tourist port [72] with the lowest levels found near rural areas. They used μ-Raman to characterize MPs, identified PP, PE, PS, PVC, and PC, and reported that 76% of suspected MPs were true polymers [71]. Authors also identified PP, PE, and PS and reported that 86% of suspected MPs were true polymers [72]. Others used μ-FTIR to identify EPS, PE, and PP MPs, and used GCMS to find polycyclic aromatic hydrocarbons (PAHs) associated with the MPs in levels that averaged 358 ng/g showing that MPs can become vectors of chemicals in water [73]. Seasonal variations of MFPs were studied in six reservoirs where slight changes in PET, PP, PS, PA, PE, PVC, and PC MPs’ levels were measured during the dry and wet seasons [65].

Vertical distributions of MPs in reservoirs were also studied [74]. The highest levels were found in the mid-depth samples and using Raman spectroscopy, authors identified PA, PVF, and PE in the surface, PA, PE, and PVF in mid depth, and MPs denser than water like PVC in the bottom. This is important to consider when explaining vertical distribution of MPs of different nature in freshwater bodies like lakes, reservoirs, of stagnant zones of water streams. Also, the MP levels in tributaries were up to six times greater than the levels in the reservoir; the polymers identified were PA, PE, PP, PVC, PVF and PS.

The distribution of MPs in a stormwater retention pond and the potential to contaminate other water bodies was studied in Denmark [75]. Implementing a QA/QC protocol, they used FPA-μ-FTIR to fully characterize MPs and identify PEST and PP as the dominant polymers. They expressed MP levels in terms of number (PEST) and mass (PP -using its density) per cubic meter of water.

### Snow and surrounding streams, and groundwater

Levels of up to 1.2 × 10^5^ MFPs/m^3^ in snow and up to 2 × 10^3^ MPs/m^3^ in streams were found at Mount Everest [76]. Authors found predominantly MFs in snow samples, which most likely came from clothes, gear, and other materials mountain climbers use. It appears directly correlated to increased tourism. The polymers identified, PEST, PMMA, nylon, and PP MFPs confirmed the likely sources. The occurrence of MFPs in groundwater was studied in three private wells (PWs) and 14 springs in a region of Karst geologic formation [77]. Authors reported levels of up to 4.4 × 10^3^ MFs/m^3^ (4.4 MFs/L) in PWs and 1.5 × 10^5^ MFs/m^3^ (150 MFs/L) in springs. Using py-GCMS, 20% of suspected MFs were identified as PE (true polymer), but level was not reported. Other MFs were lost due to combustion, a common issue when using py-GCMS when characterizing MNPs and MFs.

### Sources and fate of MNPs in water

#### Wastewater treatment plants’ effluents and removal levels

We summarized data from studies focused on characterizing MFPs in effluents from WWTPs and estimating their removals. Complete details about sample volume, number of samples, treatment process(es), MFP levels found, polymers identified, and other data extracted are shown in Table S4. Removal rates of MFPs varied depending on the treatment processes of WWTPs; MFPs of different polymer composition are still launched to the environment daily contained in the effluents, mixed sludge, or in processed heat-dried sludge marketed as soil amendment. Findings confirm WWTPs as one of the main sources of MFPs into natural water systems.

Authors found that despite estimated removal efficiencies of MFPs > 90% by WWTPs, large amounts of them are still discharged into adjacent rivers. Some estimated daily discharges of millions of MPs and MFPs into receiving waters, >6.5 × 10^7^ MPs by a secondary WWTP [78], an average of 2.2 × 10^7^ MFPs by a tertiary WWTP [2], ~6.7 × 10^6^ MPs by a conventional activated sludge plant [79]. They identified a mix of multiple polymers using FTIR and found that 39% [78] and 46.6% [79] of the suspected MFPs were true polymers. Others estimated a removal of 72% of microbeads by a WWTP but 7.4 × 10^8^ of them were discharged via the effluent daily [80]. Authors identified PP in microbeads of body scrubs and PE in microbeads of toothpastes, and PMMA resin in both. Through SEM observations, they found that ~99% of the microbeads were not damaged by enzymatic digestion and recommended that sample digestion processes are reported for a more comprehensive interpretation of results.

Authors also used μ-FTIR to characterize MFPs and estimate their removal by a conventional WWTP [81], one real-scale and one pilot-scale WWTPs of different treatment technologies [82], and five WWTPs [63]. Again, multiple polymers were identified; some were common like PE, PP, PEST, and PMMA in two of those studies [63,81] in which estimated removals were also similar, >94%. However, daily loads of MFPs entering receiving waters remained in the millions, 3.0 × 10^8^ [81] and 8.2 × 10^6^ [63]. In the real and pilot scale WWTPs studied [82], similar MFPs removals were estimated, >85%, but PUR, EPM, PE and PESTs were the polymers identified in the real scale plant and only PEST in the pilot-scale plant, which would need further assessment to explain those findings.

The impact of WWTPs effluent discharges into adjacent lakes was assessed by characterizing MFPs of different size using μ-FTIR and μ-Raman. Some characterized MFPs in effluents of three WWTPs and identified cellulose, PET, PE, PVC, PP and PU. They estimated that MF levels in a nearby lake were 100 times lower than levels in the WWTPs’ effluents [47], which explains that dilution of MFPs concentrations entering receiving waters occurs. Others characterized MPs > 20 μm by μ-FTIR and MFs < 20 μm by μ-Raman and identified PE of high and medium densities, PET, and PMMA in MPs [83]. They also found that most MFs were of natural origin and identified PEST as the synthetic MF. The identification of PE, PP, and PS in effluents of domestic and industrial WWTPs with similar MP levels using μ-Raman was reported [84]. The range of MPs’ removals by the domestic WWTPs varied widely, 35% to 98%.

To enhance the accuracy of the levels of MFPs and microbeads reported in the discharges above and to express them as number and/or mass per volume requires more detailed data. It is important that the flow of those discharges, types of polymers and their densities, and the percent of true polymers are also reported.

#### Developing sampling and analytical methods to physically and chemically characterize MNPs

In the last decade, the physical and chemical characterization of MNPs in environmental and biological samples has been challenging. The enhanced understanding of human exposures to MNPs in the environment and their potential health effects requires the accurate characterization and quantification of MNPs. Recently, a Columbia University research group developed a hyperspectral stimulated Raman scattering imaging platform to analyze NPs in bottled water with high chemical specificity and throughput [85], which is promising. For this review, however, we summarized data from earlier efforts to characterize MNPs in water samples that can lead to reliable results.

Hendrickson et al. [86] fully characterized MPs in samples of a lake. They combined microscopy, py-GCMS, and FTIR to reliably quantify MNPs in number and mass and identify the polymers. Schmidt et al. [87] assessed the short-wave infrared imaging spectroscopy for identifying MNPs in natural waters. Proximity to urban areas, WWTPs, and extreme precipitation events were likely risk factors for MNPs contamination. To identify MNPs in natural waters receiving WWTPs effluents, they recommended using both μ-FTIR and μ-Raman depending on the size of the MNPs to test for.

Nakamura et al. [88] used polyhedral oligomeric silsesquioxane networks to show that water dispersions produce bimodal light-emission bands in the blue and yellow regions. MNPs (50 nm–1 μm) of PS, PLA, and PMMA enhanced emission bands in the blue region. The NPs affected optical properties detected by drastic changes in the color of light from yellow to blue. Asamoah et al. [89] developed an optical sensor to monitor for PET MPs via laser light scattering detection from flat or curved rough (aging) and pristine MPs. The sensor can determine size, type, curvature, transparency, and translucency of MPs in water, but cannot determine concentrations.

Piarulli et al. [90] developed a near IR hyperspectral imaging technique to identify polymers in MPs ≥ 80 μm in water. Samples need no pre-sorting step, so procedural steps, analysis time, and costs are reduced. It suggests this method can be cost-effective and robust for monitoring MPs in water. Cho et al. [91] developed an analytical scheme for instantaneous capture of MPs dispersed in water applicable to field work. It includes a simultaneous one-step spectral acquisition. Using perfluoro-hexane (PFH), they captured PE MPs (300 μm). A wide area illumination Raman scheme provided a laser illumination diameter of 6 mm. In average, a droplet of PHF in tap water can recover 95.9% of PE MPs.

Efforts to enhance and standardize sampling and sample preparation methods were also carried out [92]. They assessed the cost-effectiveness of digestion methods to isolate MPs of LDPE, HDPE, PP, PS, PVC, and PET. Peroxide oxidation with zinc chloride was the most effective digestion method that led to reliable polymer identifications by μ-FTIR. In 2020, an efficient, low-cost bulk water (single pot) grab sampling method to determine MPs in water was developed [93]. The collection vessel is used for sample preparation prior to MPs analyses. It minimized contamination, degradation, and losses, while increased recoveries and throughput when compared with sieving.

Mari et al. [94] validated a Small Volume Glass Separator and procedures for sample preparation to characterize MPs in freshwater and wastewater samples using μ-FTIR. They achieved recoveries up to 39% greater than other sample preparation methods. A workflow to harmonize sampling and analytical protocols for MPs ≥ 5 μm in clean water samples was developed [95]. Researchers used a portable filtration cascade unit (pore sizes 100, 20, 5 μm) to filter tap and groundwater. Using a semi-automated μ-Raman, they identified PE more frequently than PET, PP and PA with 81% recovery in MPs < 20 μm.

Yuan et al. [96] compared the performance of in-line and in-lab filtration methods by assessing MPs recovery in samples from a tap of a DWTP that included ultrafiltration. In-line filtration yielded recoveries greater than the reference materials used, PVC, PET, and nylon. Thus, in-line filtration can help standardizing sampling methods and preventing MPs contamination during drinking water samples preparation. Others pioneered the study of protein corona-mediated extraction of NPs from natural waters [97]. The researchers used py-GCMS for NPs mass determination and polymer identification. Strict controlled physical-chemical parameters were implemented and recoveries of PS and PMMA NPs extracted from real samples varied from 72.1 to 98.9%.

#### Potential human health effects/toxicity of MNPs

Findings of studies in early 2020s revealed the presence of MNPs in human tissues, fluids (including the excretory system), organs, and feces. MPs have been found in lung tissue [98], suggesting inhalation as the exposure route, and in blood [99] suggesting several exposure routes. MNPs may affect tissues via direct physical effects (particle toxicity) or through chemical toxicity as vectors of exogenous hazardous substances and pathogens. However, accurate determinations of human exposure to MNPs to assess health effects are still poorly understood. Epidemiologic studies can help in better understanding both exposures and health effects. The Marfella et al. [100] study seems to be a first step in that direction despite its limitations. Therefore, we focused on laboratory studies aimed at better understanding how exposures to MNPs may affect human health.

We summarized data from 10 laboratory studies focused on potential health effects of MNPs or their additives on humans (Table 4). The evidence that MNPs can cause toxic effects on cells and affect inflammatory processes is growing. Choi et al. [101] demonstrated that PS MPs fragments can have chemical and physical effects on cells. The rough and sharp edges of the MPs influenced physical cytotoxicity, which correlated with direct physical damage to cell plasma membranes. Fibroblasts and red blood cells exposed to the MPs showed membrane damage and hemolysis, releasing lactate dehydrogenase and hemoglobin. MP exposures increased acute inflammation in immune-related mononuclear cells and induced oxidative stress via production of reactive oxygen species demonstrating their chemical effect. Also, the MPs induced cell death of fibroblasts and cancer cells, likely via the release of PS’s chemical additives.

Ju et al. [102] demonstrated the spontaneous binding of PVC MPs with human serum albumin driven by electrostatic forces. This binding appeared to alter the α-helix and secondary structure of albumin molecules. This suggests that PVC MPs could bind with albumin and alter its physiological function to potentially cause toxicity in vivo.

The effect of MNPs entering the digestive tract via ingestion on certain digestive and inflammatory processes was also studied. Tan et al. [103] found reduced lipid digestion in a simulated human digestive tract following exposure to various reference materials. The highest level of inhibition was associated with PS, but decreased lipid digestion was also observed with PET, PE, PVC, and PLGA. Greater PS MNPs concentrations, independent of size, were associated with larger inhibition of lipid digestion and they adsorbed and reduced the enzyme activity.

Busch et al. [104] used an in vitro model with three human cell lines representing enterocytes, mucus-producing goblet cells, and macrophages to study the role of intestinal inflammation in modulating MNP toxicity. The triple-culture models were exposed to PS and PVC MNPs for 24 h and the healthy intestine model showed no acute toxic effects. In the inflamed intestine model, exposure to PVC increased the release of IL-1β and caused a loss of epithelial cells. Therefore, preexisting intestinal inflammation such as inflammatory bowel disease should be considered when assessing the potential toxicity of ingested MNPs. Busch et al. [105] also studied the toxicological effects of buoyant PE MNPs (diameters 200–9900 nm) on cells of the healthy and inflamed intestine using an inverted in vitro triple-culture model. Changes in healthy triple cultures were not statistically significant, except for an increase of ~25% in IL-8 secretion after treatment with 50 μg/cm of PE MNPs. In inflamed triple cultures, changes were not statistically significant also but an increasing dose-dependent trend of IL-6, IL-8, and TNF-α secretion after PE exposure was observed.

Plastic additives used during plastic production and substances that can adsorb on MNPs in the environment were the focus of other studies. Additives included plasticizers (e.g., phthalates and adipates), antioxidants (e.g., phenols), and pigments (e.g., metals, benzophenones). Certain resins use BPA, which can remain in plastic products and can leach into water. Additives such as phthalates and BPA have been linked to endocrine disrupting effects in humans.

Hwang et al. [106] assessed the effects and potential toxicity of secondary PP MPs on various cell types. Cellular responses included cytotoxicity, hemolysis, release of inflammatory cytokines, histamine release, and production of reactive oxygen species. Although the MPs showed low cytotoxicity overall, MPs < 20 μm dissolved in dimethyl sulfoxide media at 1 mg/mL concentrations were cytotoxic to mononuclear cells probably due to release of chemicals and increased production of reactive oxygen species. Also, immune cells exposed to PP MPs produced more histamine and inflammatory cytokines such as IL-6 and TNF-α.

He et al. [107] evaluated cytotoxic effects of PS NPs with various surface functional group environmental transformations on human hepatocellular carcinoma (HepG2) cells in vitro. They found that more PS-COOH and PS-NH2 were internalized into HepG2 cells than PS NPs. Greater cytotoxicity and oxidative damage in HepG2 cells occurred following exposure to PS-COOH and PS-NH2 NPs of 50 nm diameter, compared to PS NPs, at concentrations of 10, 50, and 100 μg/mL. Exposure to 50 μg/mL of PS NPs yielded maximum upregulation of glutathione (GSH) content, while exposure to 10 μg/mL of PS-COOH NPs yielded maximum upregulation of superoxide dismutase (SOD) activity. However, 100 μg/mL of PS-NH2 NPs exposure yielded maximum accumulation of malondialdehyde, and reduced SOD activity and GSH content. Thus, exposure to greater levels of PS-NH2 NPs may impair antioxidant capacity and increase susceptibility to oxidative damage. Bolivar-Subirats et al. [108] studied the seasonal variability and ecotoxicity of 21 compounds associated with plastic pollution in water. They detected diethylhexylphthalate (DEHP), DEP, di-*n*-butyl phthalate (DBP), nonylphenol (NoP), octylphenol (OP), benzophenone (BP), and bisphenol A (BPA) in a river basin. Greater levels were detected near the river’s mouth, an area of higher urban density and WWTP’s discharges, during the summer. Authors used *Daphnia magna* to determine the median effective concentration, EC_50_, of the contaminants. They calculated risk quotients and found that DEHP, NoP, and OP could produce acute adverse effects.

Sixto et al. [109] studied the potential for release of phthalates and BPA from certified reference material of LDPE pellets, 110 μm, and their bioavailability within the digestive tract. They used two in vitro extraction tests representative of the dynamic physicochemical conditions of the human gastrointestinal (GI) tract and detected releases of BPA, dimethylphthalate, and diethylphthalate. Average bioaccessibility values spanned in range from 51 to 87% for both methods. Findings contributed with developing extraction methods for the determination of oral bioaccessibility of phthalates and BPA pools in LDPE MPs.

Trujillo-Rodriguez et al. [110] compared sample preparation methods to determine bioaccessible endocrine disrupting chemicals (EDCs) in the gastric (G) and GI phases of the unified bioaccessibility method (UBM). EDCs dissolve upon ingestion, are available for absorption, and may pose risks to human health. Researchers assessed bioaccessibility pools of compounds leached from beach sand contaminated with PE MPs, including dialkyl phthalate congeners and BPA. Important to consider that BPA has been found in freshwater bodies. Significant releases of dimethyl phthalate, diethyl phthalate, and BPA from the sample into G and GI fluids occurred during digestion at low pH. Risk assessment suggested potential concentration of the phthalates and BPA in body fluids. Average daily intake values corrected with bioaccessibility factors made authors determine that exposure to phthalates and BPA in MPs-laden beach sand do not pose a significant health risk for children.

## 4. Conclusions

Forty polymers were reported in the 105 papers we selected. We did not find direct evidence of human exposure to MNPs in water, but all those polymers were reported in the papers selected for this review. Although polymers are not considered toxic to humans, they can break down and leach chemicals like additives used during plastic production, which can be harmful to humans when exposed. For example, vinyl chloride from PVC can harm the liver, nervous system, and lungs and cause a variety of cancers in organs like the liver, brain, and lungs [102]. Similarly, BPA, commonly used in plastics manufacturing, is a known endocrine disruptor linked to harmful health effects in humans [108]. Thus, evidence that direct exposure to MNPs can harm human health is growing.

There is a need for formulating and describing criteria to ensure test reliability for quantifying MNPs and for physically and chemically characterizing them. Combining microscopic methods, including electron microscopy (i.e., SEM and transmission electron microscopy), and spectroscopic methods or py-GCMS could help in accurately counting and fully characterizing MNPs. Understanding them as multi-dimensional particles is important. Reliable MNPs characterization is needed to accurately assess human exposure to MNPs and determine potential harmful health effects. Thus, analytical methods that can quantify and fully characterize MNPs in water and other environmental samples are needed. However, sample collection and preparation, potential water interference during analytical work and limits of detection, lack of reference materials for most polymers found in water, and laboratory expertise are some of the limitations that play a role in the planning of studies pursuing the production of reliable results. Addressing those limitations could better inform the comprehensive interpretation of results. It becomes even more important when planning exposure and risk assessment studies.

Making conversions of MNPs concentrations from number per volume to mass per volume possible, like what [75] reported, and vice versa is important because MNPs could harm health via physical and biochemical mechanisms. Depending on the stiffness and edge characteristics, MNPs could physically harm cells. And, depending on their chemical composition they could act as toxic agents. Also, they can function as vectors when adsorbing chemicals and/or microorganism present in water. Taking the above into account during the planning and design of exposure and risk assessment studies becomes of upmost importance. However, reliable quantitative and qualitative results are needed. Some of them include the number, shape, and dimensions of MNPs (shape and dimensions allow to estimate MNPs’ volumes), and density of the polymer. All of it requires a careful planning given current limitations to accurately count MNPs and fully characterize them.

Regarding studies that involve drinking water and wastewater, detailed descriptions of the treatment processes and the distribution networks or discharges to the environment are needed. Current DWTPs and WWTPs were not designed to remove MNPs, and these descriptions could help identify potential entry points for MNPs into the treatment systems or deficiencies along treatment stages. In the case of DWTPs, descriptions would also provide information on potential MNPs’ contamination within the distribution networks and plan interventions to prevent potential harmful exposures. Furthermore, findings of laboratory-scale studies are showing enhanced MNPs’ removal that could benefit real scale DWTPs.

Currently, due to the lack of standard sampling protocols and analytical methods, we cannot directly compare MNPs in all types of water samples found in the published work [111]. It is essential that we tackle this gap because without accurate and reliable data, efforts to protect both the environment and public health will not be fruitful. These challenges are not easy to fix. Efforts to develop suitable technologies and methods that are robust, relatively inexpensive, and not too complex to use for sampling and fully characterizing MNPs in water are currently underway. In so doing, we will continue working to better protect our communities, now and in the future.

## Supplementary Material

PRISMA2020-Checklist-SuppDocs2

SupportingDocs1-Tables S1-S4

**Supplementary Materials:** The following supporting information can be downloaded at: https://www.mdpi.com/article/10.3390/microplastics4030060/s1, Supporting Documents 1: Table S1: Search Query; Table S2: MNPs in rivers; Table S3: MPs in lakes, reservoirs, and retention ponds; Table S4: MPs in effluents of wastewater treatment plants. Supporting Documents 2: PRISMA 2020 check list.

## Figures and Tables

**Table 1.** MNPs in drinking water systems

				Concentration (MPs/L, unless otherwise indicated)			
Author	Number of sampling sites/points	Number of samples	Sample volume {sampling point] (method)	Mean	Range	Polymer	Polymer identification method
**Tap water**							
Kankanige et al. (2020)	5	45	1 L (gb)	6 ± 3 (>500 pm); 56 ± 14 (6.5–53 μm)		PE > PVC > PET, PA, PP, PAM, PTFE, PS, PMMA (both sizes)	ATR-FTIR
Shruti et al. (2020)	42	126	1 L (gb)	0.018 ± 0.007	5 ± 2 to 91 ± 14	PTT, epoxy resin	μ-Raman
Tong et al., (2020)	38	76	1 L (HDPE b)	440 ± 275	0 to 1.25×10^3^	PE ~ PP > PE/PP > PPS ~ PS > PET	μ-Raman
Almaiman et al. (2021)	2	4	1 L		0 to 1.8	PE	μ-FTIR
Feld et al. (2021)	17	20	50 L (s-s sc)	0.02		PET, PP, PS, and ABS	μ-FTIR
Weber et al. (2021)	9	18	0.25–0.5 m^3^ [TS & HC]; 0.5–1.5 m^3^ [tap] (v-r s-a)	0	0	N/A	μ-Raman
**Water source (raw water) to treated, distribution system, and drinking or tap water**							
Pivokonsky et al. (2018)	3 / 6	72	1 L (DW)		338±76 to 628±28 (DW)	PET > PP, PAM (TPs), and PVC (TP2); PE > PET > PP, PAM (TP3)	FTIR and μ-Raman
Mintenig et al. (2019)	23	23	300–1,000 L [raw]; 1,200–2,500 L [tr]		0 to 0.001 (tr); 0 to 0.003 (meter)	PEST >> PA > PVC (2 tr; 4 HH meter)	μ-FTIR
Pivokonsky et al. (2020)	2 / 8	48	2 L		14 ± 1 (TP-M); 151 ± 4 (TP-P)	Cellulose acetate > PE, PET > PVC, PBA, PTFE (TP-M); cellulose acetate, PE, PET, PP, PS, PVC (TP-P)	μ-Raman
Zhang et al. (2020)	14 (7 tap; 7 raw)	42	4.5 L [tap] (gb); 50 L [raw]	7×10^2^±6×10^2^ (tap); 4×10^2^±3×10^2^ (raw)	0.3 – 1.6 (tap)	Rayon > PET > PE (tap); PET > Rayon > PE, PVC, PEST (raw)	μ-FTIR
Chanpiwat et al. (2021)	6 (4 TPs; 2 canals) / 14 (8 TPs; 6 canals)	28	~100 L		0.44 – 1.00 (tap-E); 0.24 – 1.00 (tap-W)	PE > PET/PP > PS > PVC (tap); PE, PET > PP > PS, PVC (canal)	μ-Raman
Kirstein et al. (2021)	5	15 (ds-1); 14 (ds-2)	200 – 1,100 L	0.174 ± 0.405 (ds)	19 ± 14 to 809 ± 688 (0.004 ± 0.007 to 2.6 ± 3.0 ng/L)	PEST (ds-1 pump, ds-2 hydrant); PA (ds-2 pump, ds-1 hydrant). Acrylic, PVC, PS	μ-FTIR and py-GCMS
Shen et al. (2021)	3	12	30 L	2.75×10^3^ (raw); 3.52×10^2^ (tr); 3.44×10^2^ (tap)	3.4×10^2^ to 4.0×10^2^ (tr); 2.7×10^2^ to 4.0×10^2^ (tap)	PET, PE > PP >>> PA (tr); PE, PET > PVC, PP >>> PA (tap)	μ-FTIR
Chu et al. (2022)	5	10	1 L	13.2 (tap); 95.6 (tr)		PEST > PS, nylon (tap); PEST > PP, PVC, nylon (tr)	μ-FTIR
**Removal efficiency**							
Johnson et al. (2020)	16	69	0.18 m^3^ (raw); 19.88 m^3^ (tap)	1.5×10^4^ MPs/m^3^ (raw, >LOQ)	0.001 – 0.024 (tap, >LOD but <LOQ)	ABS, PA, PE, PET, PP, PMMA, PS, PVC, PU (>LOD, <LOQ; tap); PE, PET, PP (raw)	μ-FTIR
Dalmau-Soler (2021)	1 / 5	19	2.5 L (raw; grab); 50 L (sf & GAC) and 100 L (RO & final) [sieves]		0.06±0.04 (tr); 9.6×10^2^±4.6×10^2^ MPs/m^3^ (raw)	PES > PP (tr); PES > PE (raw)	μ-FT-IR
Gomiero et al. (2021)	4	12	1 m^3^	6.1 (tr) – 93.1 (raw) pg/m^3^ or [6.1 – 93.1 ng/L]		PE >> PA ~ PET (ds)	py-GCMS
Kankanige & Babel (2021)	1 / 5	10	10 L (gb)	Dry: 4.5×10^2^ (tr); 1.4×10^3^ (raw). Rainy: 7.7×10^2^ (tr); 1.8×10^3^ (raw)		PE, PP, PVC, PET, PS (tr)	ATR-FTIR and confocal Raman
Radityaningrum et al. (2021)	2 / 14	28	2.5 L (grab)	TP 1: 3.1 (tr); 6.6 (raw). TP 2: 2.1 (tr); 8.8 (raw)		Cellophane (tr)	FTIR

**Note.** distribution system (ds), drinking water (DW), glass bottle (gb), HDPE bottle (HDPE b), house connection (HC), household (HH), stainless-steel sampling column (s-s sc), transfer station (TS), treated (tr), treatment plant (TP), volume-reduced sampling apparatus (v-r s-a)

**Table 2.** Removal of MNPs during DW treatment processes – Laboratory-scale studies

Author	Number of samples	Sample volume	Removal (%)	Polymer	Polymer identification method
Chen et.al. (2021)	6 (Mg/Al ratios); 8 (pH)	n.a.	>90	PS	UV–vis spectrophotometer and FTIR
Zhang et.al. (2021)	21 (7 different dosages of coagulant aid)	n.a.	54.70% (ASA-conv dose); 91.45% (PAM high dose)	PET	FTIR, XPS
Li et.al. (2022)	n.a.	n.a.	99.9% MW degradation; 42.7% mineralization (O_3_)	PS	FTIR, XPS
Shi . (2022)	84 (7 different dosages of nano-Fe_3_O_4_ for each of 4 MPs -triplicate)	300 mL	86.87± 6.92% (PE); 85.05±4.70% (PP); 86.11±6.21% (PS); 62.83±8.34%. (PET)	PE, PP, PS, PET	SEM; μ-FTIR

**Note.** Not available (n.a.)

**Table 3.** MNPs in tap water, bottled water, and water-based beverages

			Concentration (MPs/L)			
Author (Year)	# Samples	Sample volume	Mean	Range	Polymer	Polymer ID method

Kosuth et al., (2018)	156 (tap); 3 (b); 12 (be)	0.55 L (tap); 1 L (be); 1 L (tap-be)	3.57±1.79 (b); 4.05 (be)	3.23±3.48 – 9.24±11.80 (tap); 1.78 – 5.37 (b); 0 – 14.3±3.21 (be)		
Mason et al., (2018)	259	0.5–0.6 L; 0.75 L; 2 L	10.4 (>100 μm); 325 (6.5 to >100 μm)		PP > nylon > PS, PE > PEST	ATR-FTIR
Oßmann et al., (2018)	32	0.25 L	3,074±2,531	2,649 (su); 6,292 (g)	PET (su); PE > PP (g)	μ-FTIR
Schymanski et al., (2018)	114 (triplicate)	0.7 – 1.5 L	14±14 (su); 118± 88 (rp); 50±52 (g); 11±8 (c)	2–44 (su); 28–241 (rp); 4–156 (g); 5–20 (c)	PEST > PE > PP, PA (su); PEST > PP, PE > PA (rp); PEST, PE > PA > PP (g); PE > PEST > PP (c)	μ-Raman
Kahle 2021	10	1 mL (w); 150 mL (be)			PVPP (filter stabilizer)	μ-Raman ATR-FTIR and confocal Raman
Kankanige and Babel, (2020)	65 (su); 30 (g)	2.4 – 6.0 L (su); 3.3 L (g)	140±19 (su); 52±4 (g)		PET > PE > PP >> PA > PVC	
Shruti et al., (2020)	57	1 L		1±1 – 6±2 (tea); ND to 7±3.2 (Sd & Ed); ND to 28±5.3 (be)	PA > PEA (all); ABS (Ed); PET (be)	μ-Raman
	4	18.5 L and 19 L		<LOQ to 26	PE, PS	
	4	1.5 L		<LOQ		
	8	0.6 L	1.6×10^3^ (PE); 1.7×10^3^ (PA)		PE, PA	
Almaiman et al., (2021)	12	0.5 L		2	PET	μ-FTIR
	6	0.4 L		<LOQ		
	88	0.33 L		0.99 to 4		
	24	0.30 L		<LOQ		
	46	0.20 L	1.2 (PP); 1.3 (PET)			
Weisser et al., (2021)	4	0.7 – 0.75 L	295 (f&c)	317±257	PE >> PS > PVC > PA > PEST	ATR-FTIR and μ-FTIR
Li et al., (2021)	8	0.18 L		1.31×10^6^ to 1.62×10^7^		Raman
Song et al., (2021)	3 FBs; 4 WBs			1.0×10^4^ to 1.12×10^5^ (FBs); 1.3×10^4^ to 3.7×10^4^ (WBs)	PPSU (FBs); PC and PP (WBs)	μ-FTIR
Winkler et al., (2019)	3		BCs 1–3: 6.3×10^4^; 1.2×10^6^; 3.3×10^5^		HDPE, PET	EDS
Hernandez et al., (2019)	12 (3 per teabag)	0.01 L	2.3×10^6^ (>1 μm); 1.5×10^10^ (<1 μm)		Nylon, PET (releases from empty tea bags)	ATR-FTIR

**Note.** Beer (be), bottle (b), bottle cap (BC), carton (c), energy drink (Ed), feeding bottle (FB), filled & capped bottle (f&c), glass (g), level of quantification (LOQ), non-detect (ND), returnable plastic (rp), single-use plastic (su), soft drink (Sd), water (w), water bottle (WB)

**Table 4.** Summary of scientific publications selected by toxic effects of MNPs on human cells, year, and country (number)

Topic	Year	Country (n)	References
Toxicity studies of micro and nanoplastics and plastic additives on human cells	2019	South Korea (1)	Hwang, Choi et al. (2019)
	2020	China (2), South Korea (1), China-United States (1)	He, Li et al. (2020), Ju, Zhang et al. (2020), Choi, Bang et al. (2020), Tan, Yue et al. (2020)
	2021	Germany (2), Spain (3)	Busch, Bredeck et al. (2021), Busch, Kampfer and Schins (2021), Bolivar-Subirats, Rivetti et al. (2021), Sixto, El-Morabit et al. (2021), Trujillo-Rodriguez, Gomila et al. (2021)

## Box 1. Acronyms of polymers reported in selected papers.

ABS: acrylonitrile butadiene styrene

EP: ethylene-propylene copolymer (EPM and EPDM also used)

EPR: ethylene-propylene rubber (also known as. ethylene-propylene diene monomer, EPDM)

EPS: expanded polystyrene

EVA: ethylene-vinyl acetate (also known as. poly(ethylene-vinyl acetate), PEVA)

PA: polyamide (a family of amide linked polymers)

PAEK: polyaryletherketone

PAM: polyacrylamide

PAN: polyacrylonitrile

PBMA: poly(butyl methacrylate)

PBT: poly butylene terephthalate

PC: polycarbonate

PE: polyethene

PEA: poly(ester-amide)

PEP/PED: peptoid polymers

PEST: polyester (Pest and PES also used)

PESU: polyethersulfone (also commonly abbreviated as PES)

PF: phenol-formaldehyde resin(s)

PET: poly(ethylene terephthalate; PEP also used)

PLGA: poly(lactic-co-glycolic acid)

PMMA: poly(methyl 2-methylpropenoate), poly methyl methacrylate, or acrylic

PMS: poly alpha-methylstyrene

POE: polyolefin elastomer(s)

POM: polyoxymethylene

PP: poly(1-methylethylene) or polypropylene

PPS: polyphenylene sulfite

PPTA: poly(p-phenylenterephthalamide) -trade name Kevlar

PS: poly(1-phenylethene-1,2-diyl) or polystyrene

PTFE: polytetrafluoroethylene

PTT: poly-trimethylene terephthalate

PUA: polyurethane-acrylate(s)

PVA: polyvinyl acetate

PVC: poly(1-chloroethylene) (also known as. poly vinyl chloride)

PVC-U: unplasticized PVC

PVDC: polyvinylidene chloride (also known as. polyvinylidene dichloride)

PVF: polyvinyl fluoride

PVPP: polyvinylpolypyrrolidone (also abbreviated as PVP and also known as. povidone)

PVS: polyvinyl siloxane (also known as. poly-vinyl siloxane, vinyl polysiloxane (VPS))

PU: ethyl urea also known as. polyurethane (PUR also used)

UF: urea-formaldehyde
