# Supplementary material for: Human Exposures to Micro- and Nanoplastics in Water and Data Needed to Understand Potential Health Effects—A-State of the Science Review
Source: Microplastics. Author manuscript; Available in PMC 2026 Apr 8. (PMC13055644; doi:10.3390/microplastics4030060)
Supplement: PRISMA2020-Checklist-SuppDocs2 [file NIHMS2151437-supplement-PRISMA2020-Checklist-SuppDocs2.pdf]

PRISMA 2020 Checklist

Section and Topic	Item #	Checklist item	Location where item is reported
**TITLE**			
Title	1	Human exposures to micro- and nanoplastics in water and data needed to understand potential health effects – A-state of the science review	Protocol & manuscript
**ABSTRACT**			
Abstract	2	Abstract is compliant with the 12 elements of the PRISMA 2020 checklist.	Manuscript: Abstract
**INTRODUCTION**			
Rationale	3	This is described in the top two paragraphs of the Introduction section.	Manuscript: Introduction -top two paragraphs
Objectives	4	These are stated in the bottom two paragraphs of the Introduction section.	Manuscript: Introduction - third & fourth paragraphs
**METHODS**			
Eligibility criteria	5	The inclusion and exclusion criteria for the review and how the studies were grouped for the syntheses are described in the Literature search and selection for data extraction subsection of the Methodology section. They are also described in the protocol.	Protocol & manuscript: Methodology.
Information sources	6	All databases and reference lists searched and consulted to identify studies are described in the Literature search and selection for data extraction subsection of the Methodology section. They are also described in the protocol. Sources were last searched between February and March 2022.	Protocol & manuscript: Methodology, literature search & selection for data extraction.
Search strategy	7	The full search strategies for all databases are described in the manuscript's Methodology section, literature search and selection for data extraction subsection, and in the protocol as well.	Protocol & manuscript: Methodology, literature search & selection for data extraction
Selection process	8	The inclusion criteria of studies for the review, the number of reviewers who screened each record in the three stages of Covidence (https://www.covidence.org/#) working independently are described in the manuscript's I Methodology section, literature search and selection for data extraction subsection, and Figure 1. It is also described in the protocol.	Protocol, Covidence, & manuscript: Methodology, literature search & selection for data extraction, Fig. 1.
Data collection process	9	The methods used to extract data from studies, including the number of reviewers who extracted the data working independently are described in the manuscript's Methodology section, literature search and selection for data extraction, and Figure 1. This work is also described in the protocol.	Protocol & manuscript: Methodology, literature search

Section and Topic	Item #	Checklist item	Location where item is reported
			& selection for data extraction, Fig. 1.
Data items	10a	The findings for which data were sought and the methods used to decide the findings to collect are described in the manuscript's Methodology section, data extraction and summary subsection. They are also described in the protocol.	Protocol & manuscript: Methodology, data extraction & summary.
	10b	The type of data that were sought and assumptions made about unclear information are described in the manuscript's Methodology section, data extraction and summary subsection. It is also described in the protocol.	Protocol & manuscript: Methodology, data extraction & summary.
Study risk of bias assessment	11	In both the protocol and the manuscript's Methodology section, literature search and selection for data extraction subsection the methods to assess the inclusion of studies and number of reviewers who assessed each study working independently are described in detail. The use of Covidence during the process is also described in the section and subsection mentioned.	Protocol & manuscript: Methodology, literature search & selection for data extraction, used Covidence.
Effect measures	12	Effect measures did not apply to this review.	Not applicable (N/A)
Synthesis methods	13a	The decision-making process used to select studies for each synthesis (MNPs in drinking water and sources, wastewater effluents, etc.) are described in the protocol and the manuscript's Methodology section, literature search and selection for data extraction subsection.	Protocol & manuscript: Methodology, literature search & selection for data extraction.
	13b	Methods used to prepare the data for presentation or synthesis are described in the manuscript's Methodology section, literature search and selection for data extraction subsection.	Manuscript's Methodology, literature search & selection for data extraction.
	13c	Methods used to tabulate or visually display results of individual studies and syntheses were Covidence to prepare the PRISMA flowchart (Figure 1), Tableau (Figure 2), and Excel (Figure 3 and all the Tables).	Covidence's PRISMA flowchart (Fig. 1); Tableau (Fig. 2); Excel (Fig. 3 and all Tables).
	13d	Meta-analysis and statistical heterogeneity analysis did not apply to this review.	N/A
	13e	Exploring possible causes of heterogeneity among study results did not apply to this review	N/A
	13f	Sensitivity analyses did not apply to this review. MNPs are emerging environmental contaminants of concern with no standard methods for	N/A -emerging

Section and Topic	Item #	Checklist item	Location where item is reported
		sampling and testing.	contaminants with no standard methods for sampling & testing.
Reporting bias assessment	14	Assessing risk of bias due to missing results in a synthesis did not apply to this review. MNPs are emerging environmental contaminants of concern with no standard methods for sampling and testing.	N/A -emerging contaminants with no standard methods for sampling & testing.
Certainty assessment	15	The methods used for assessing confidence in the body of evidence for findings of MNPs in water of different matrices are described in the manuscript's Findings and discussion section. Reviewers focused on the quality description of the sampling and analytical methods.	Manuscript - Findings & discussion - description of sampling & analytical methods.
**RESULTS**			
Study selection	16a	The description of number of studies selected for the review is given by the PRISMA flow diagram (Figure 1) that was generated via Covidence during the review process.	Manuscript: Fig. 1 (Covidence's flowchart).
	16b	Studies that apparently met the inclusion criteria were excluded because their sampling and/or analytical methods lacked a detailed description. It is described in the manuscript's Findings and discussion section.	Manuscript - Findings & discussion.
Study characteristics	17	Characteristics of each included study are presented in the manuscript's Findings and discussion section, and they are included in all the Tables.	Manuscript - Findings & discussion.
Risk of bias in studies	18	Assessments of risk of bias for each included study were not applicable. MNPs are emerging environmental contaminants of concern with no standard methods for sampling and testing.	N/A -emerging contaminants.
Results of individual studies	19	(a) A summary statistic for each matrix of water in which MNPs were reported is found in the manuscript's Findings and discussion section and in all the Tables of the manuscript. And (b), effect estimates are not applicable to this project	(a) Manuscript - Findings & discussion, including Tables; (b) N/A.
Results of syntheses	20a	The characteristics and risk of bias among contributing studies did not apply to this review because MNPs are emerging contaminants with no standard methods for sampling & testing.	N/A -emerging contaminants with no standard methods for sampling & testing.
	20b	No statistical syntheses were applicable to this review; and no meta-analysis was conducted. MNPs are emerging environmental	N/A -emerging

PRISMA 2020 Checklist

Section and Topic	Item #	Checklist item	Location where item is reported
		contaminants of concern with no standard methods for sampling & testing.	contaminants.
	20c	No possible causes of heterogeneity among study results apply to this review. MNPs are emerging contaminants of concern with no standard methods for sampling & testing,	N/A -emerging contaminants.
	20d	Sensitivity analyses did not apply to this review. MNPs are emerging contaminants of concern that lack standard methods for sampling and testing, which is a barrier for sensitivity analyses.	N/A -Manuscript, Findings & discussion.
Reporting biases	21	Biases on risk assessments did not apply. The lack of standard methods for sampling and testing is a barrier for assessing risk of bias.	N/A -Manuscript, Findings & discussion.
Certainty of evidence	22	Assessment of certainty in the body of evidence for the findings assessed was possible due to the full characterization of MNPs.	Manuscript: Findings & discussion.
**DISCUSSION**			
Discussion	23a	A general interpretation of the results in the context of other findings was presented and discussed.	Manuscript: Findings & discussion.
	23b	Limitations regarding the evidence of the full characterization of MNPs in water matrices were discussed for the review.	Manuscript: Findings & discussion, & Conclusions.
	23c	Limitations of the review processes used were discussed and presented.	Manuscript: Findings & discussion, & Conclusions.
	23d	Implications of the findings on the removal of MNPs in drinking water and wastewater treatment and the need to fully characterize MNPs in different water matrices regarding policy development and future research were discussed and presented.	Manuscript: Findings & discussion, & Conclusions.
**OTHER INFORMATION**			
Registration and protocol	24a	Although the review was not registered, both the protocol and draft manuscript were cleared by the Center for Disease Control and Prevention's National Center for Environmental Health.	Review was not registered.
	24b	The protocol was registered with PROSPERO (https://www.crd.york.ac.uk/prospero/), the ID number is CRD42021278806.	Manuscript: Methodology - first paragraph.
	24c	The protocol was amended to (a) add one review team member, and (b) update on progress.	PROSPERO's website.
Support	25	No financial support was available for the review.	N/A
Competing interests	26	The review authors have no competing interests.	N/A

PRISMA 2020 Checklist

Section and Topic	Item #	Checklist item	Location where item is reported
Availability of data, code and other materials	27	Templates of data collection forms, the data extracted from included studies, and the data used for all analyses in the review are available via the electronic platform of Covidence, which can be available in Excel format.	Covidence/Excel

From: Page MJ, McKenzie JE, Bossuyt PM, Boutron I, Hoffmann TC, Mulrow CD, et al. The PRISMA 2020 statement: an updated guideline for reporting systematic reviews. BMJ 2021;372:n71. doi: 10.1136/bmj.n71.
This work is licensed under CC BY 4.0. To view a copy of this license, visit https://creativecommons.org/licenses/by/4.0/
