# Supplementary material for: Human Exposures to Micro- and Nanoplastics in Water and Data Needed to Understand Potential Health Effects—A-State of the Science Review
Source: Microplastics. Author manuscript; Available in PMC 2026 Apr 8. (PMC13055644; doi:10.3390/microplastics4030060)
Supplement: SupportingDocs1-Tables S1-S4 [file NIHMS2151437-supplement-SupportingDocs1-Tables_S1-S4.pdf]

Human exposures to micro- and nanoplastics in water and data needed to understand potential health effects – A state-of-the-science review

Supporting documents 1

Table of Contents

	Page(s)
Table S1. Search Query	2-7
Table S2. MNPs in rivers	8-10
Table S3. MPs in lakes, reservoirs, and retention ponds	11
Table S4. MPs in effluents of wastewater treatment plants	12

DO NOT SHARE

Table S1. Search Query

Search Strategy:

Database	Strategy	Records
**Medline (OVID) 1946-**	Microplastic* OR nanoplastic* OR micro-plastic* OR nano-plastic* OR Plastic resin* OR plastic particulate* OR plastic particle* OR plastic waste OR plastic pollution OR microbead* OR micro-bead*<br><br>AND<br><br>Water* OR soil OR pool* OR lake* OR freshwater OR river* OR stream OR streams OR ocean* OR beach* OR sea OR seaside OR rain* OR groundwater OR sewer* OR sewage OR wastewater*<br><br>AND<br><br>Ingest* OR digest* OR drink* OR swallow* OR cook* OR gastro* OR inhal* OR respirat* OR breath* OR lung* OR expos* OR consum* OR derma* OR skin OR contaminat* OR concentrate* OR health OR illness* OR disease* OR poison* OR absorb* OR absorption* OR adsorb* OR adsorption* OR (human ADJ2 cell*)<br><br>NOT<br><br>Exp animals/ NOT exp humans/	2115
**Embase (OVID) 1947-**	Microplastic* OR nanoplastic* OR micro-plastic* OR nano-plastic* OR Plastic resin* OR plastic particulate* OR plastic particle* OR plastic waste OR plastic pollution OR microbead* OR micro-bead*<br><br>AND<br><br>Water* OR soil OR pool* OR lake* OR freshwater OR river* OR stream OR streams OR ocean* OR beach* OR sea OR seaside OR rain*	2083<br><br>-1384 duplicates<br><br>=699 unique items

DO NOT SHARE

	OR groundwater OR sewer* OR sewage OR wastewater*<br><br>AND<br><br>Ingest* OR digest* OR drink* OR swallow* OR cook* OR gastro* OR inhal* OR respirat* OR breath* OR lung* OR expos* OR consum* OR derma* OR skin OR contaminat* OR concentrate* OR health OR illness* OR disease* OR poison* OR absorb* OR absorption* OR adsorb* OR adsorption* OR (human ADJ2 cell*)<br><br>NOT<br><br>Exp animal/ NOT exp human/<br><br>NOT pubmed/medline	
**CAB Abstract (OVID) 1973-**	Microplastic* OR nanoplastic* OR micro-plastic* OR nano-plastic* OR Plastic resin* OR plastic particulate* OR plastic particle* OR plastic waste OR plastic pollution OR microbead* OR micro-bead*<br><br>AND<br><br>Water* OR soil OR pool* OR lake* OR freshwater OR river* OR stream OR streams OR ocean* OR beach* OR sea OR seaside OR rain* OR groundwater OR sewer* OR sewage OR wastewater*<br><br>AND<br><br>Ingest* OR digest* OR drink* OR swallow* OR cook* OR gastro* OR inhal* OR respirat* OR breath* OR lung* OR expos* OR consum* OR derma* OR skin OR contaminat* OR concentrate* OR health OR illness* OR disease* OR poison* OR absorb* OR absorption* OR adsorb* OR adsorption* OR (human ADJ2 cell*)	2336<br><br>-1144 duplicates<br><br>=1192 unique items

DO NOT SHARE

**CINAHL (Ebsco)**	Microplastic* OR nanoplastic* OR micro-plastic* OR nano-plastic* OR "Plastic resin*" OR "plastic particulate*" OR "plastic particle*" OR "plastic waste" OR "plastic pollution" OR microbead* OR micro-bead*<br><br>AND<br><br>Water* OR soil OR pool* OR lake* OR freshwater OR river* OR stream OR streams OR ocean* OR beach* OR sea OR seaside OR rain* OR groundwater OR sewer* OR sewage OR wastewater*<br><br>AND<br><br>Ingest* OR digest* OR drink* OR swallow* OR cook* OR gastro* OR inhal* OR respirat* OR breath* OR lung* OR expos* OR consum* OR derma* OR skin OR contaminat* OR concentrate* OR health OR illness* OR disease* OR poison* OR absorb* OR absorption* OR adsorb* OR adsorption* OR (human N2 cell*)<br><br>Exclude Medline records	25<br><br>-5 duplicates<br><br>=20 unique items
**GreenFile (Ebsco)**	Microplastic* OR nanoplastic* OR micro-plastic* OR nano-plastic* OR "Plastic resin*" OR "plastic particulate*" OR "plastic particle*" OR "plastic waste" OR "plastic pollution" OR microbead* OR micro-bead*<br><br>AND<br><br>Water* OR soil OR pool* OR lake* OR freshwater OR river* OR stream OR streams OR ocean* OR beach* OR sea OR seaside OR rain* OR groundwater OR sewer* OR sewage OR wastewater*<br><br>AND<br><br>Ingest* OR digest* OR drink* OR swallow* OR cook* OR gastro* OR inhal* OR respirat* OR breath* OR lung* OR expos* OR consum* OR	1733<br><br>-1173 duplicates<br><br>=560 unique items

DO NOT SHARE

	derma* OR skin OR contaminat* OR concentrate* OR health OR illness* OR disease* OR poison* OR absorb* OR absorption* OR adsorb* OR adsorption* OR (human N2 cell*)	
**Environmental Science Collection (ProQuest)**	TI,AB(Microplastic* OR nanoplastic* OR micro-plastic* OR nano-plastic* OR "Plastic resin*" OR "plastic particulate*" OR "plastic particle*" OR "plastic waste" OR "plastic pollution" OR microbead* OR micro-bead*)<br><br>AND<br><br>TI,AB(Water* OR soil OR pool* OR lake* OR freshwater OR river* OR stream OR streams OR ocean* OR beach* OR sea OR seaside OR rain* OR groundwater OR sewer* OR sewage OR wastewater*)<br><br>AND<br><br>TI,AB(Ingest* OR digest* OR drink* OR swallow* OR cook* OR gastro* OR inhal* OR respirat* OR breath* OR lung* OR expos* OR consum* OR derma* OR skin OR contaminat* OR concentrate* OR health OR illness* OR disease* OR poison* OR absorb* OR absorption* OR adsorb* OR adsorption* OR (human NEAR/2 cell*))	2262<br><br>-2053 duplicates<br><br>=209 unique items
**Scopus**	TITLE-ABS-KEY(Microplastic* OR nanoplastic* OR micro-plastic* OR nano-plastic* OR "Plastic resin*" OR "plastic particulate*" OR "plastic particle*" OR "plastic waste" OR "plastic pollution" OR microbead* OR micro-bead*) AND TITLE-ABS-KEY(Water* OR soil OR pool* OR lake* OR freshwater OR river* OR stream OR streams OR ocean* OR beach* OR sea OR seaside OR rain* OR groundwater OR sewer* OR sewage OR wastewater*) AND TITLE-ABS-KEY(Ingest* OR digest* OR drink* OR swallow* OR cook* OR gastro* OR inhal* OR respirat* OR breath* OR lung* OR expos* OR consum* OR derma* OR skin OR contaminat* OR	2416<br><br>-809 duplicates<br><br>=1607 unique items

DO NOT SHARE

	concentrate* OR health OR illness* OR disease* OR poison* OR absorb* OR absorption* OR adsorb* OR adsorption* OR (human W/2 cell*)) AND (INDEXTERMS(human*) OR KEY(human*)) AND NOT INDEX(medline)	
**Compendex Engineering Village**	((Microplastic* OR nanoplastic* OR micro-plastic* OR nano-plastic* OR "Plastic resin*" OR "plastic particulate*" OR "plastic particle*" OR "plastic waste" OR "plastic pollution" OR microbead* OR micro-bead*) WN AB) AND ((Water* OR soil OR pool* OR lake* OR freshwater OR river* OR stream OR streams OR ocean* OR beach* OR sea OR seaside OR rain* OR groundwater OR sewer* OR sewage OR wastewater*) WN AB) AND ((Ingest* OR digest* OR drink* OR swallow* OR cook* OR gastro* OR inhal* OR respirat* OR breath* OR lung* OR expos* OR consum* OR derma* OR skin OR contaminat* OR concentrate* OR health OR illness* OR disease* OR poison* OR absorb* OR absorption* OR adsorb* OR adsorption* OR "human cell*") WN AB)	2661<br><br>-2520 duplicates<br><br>=141 unique items
**Cochrane Library**	(Microplastic* OR nanoplastic* OR micro-plastic* OR nano-plastic* OR "Plastic resin*" OR "plastic particulate*" OR "plastic particle*" OR "plastic waste" OR "plastic pollution" OR microbead* OR micro-bead*):ti,ab<br><br>AND<br><br>(Water* OR soil OR pool* OR lake* OR freshwater OR river* OR stream OR streams OR ocean* OR beach* OR sea OR seaside OR rain* OR groundwater OR sewer* OR sewage OR wastewater*):ti,ab<br><br>AND<br><br>(Ingest* OR digest* OR drink* OR swallow* OR cook* OR gastro* OR inhal* OR respirat* OR breath* OR lung* OR expos* OR consum* OR	8<br><br>-4 duplicates<br><br>=4 unique items

DO NOT SHARE

	derma* OR skin OR contaminat* OR concentrate* OR health OR illness* OR disease* OR poison* OR absorb* OR absorption* OR adsorb* OR adsorption* OR (human NEAR/2 cell*)):ti,ab	

Notes: Duplicates were identified using the Endnote automated "find duplicates" function with preference set to match on title, author and year, and removed from your Endnote library. There will likely be additional duplicates found that Endnote was unable to detect.

DO NOT SHARE

A review on human exposures to micro- and nanoplastics in water and potential health effects – Supplemental material

Table S2. MNPs in rivers

Author (year)	Sampling sites (#)	Samples (#)	Sample volume (technique)	Concentration (MPs/m^3) Mean	Concentration (MPs/m^3) Range	Polymer(s)	Polymer ID method
McCormick et al. (2016)	9	18	N/A (neuston net 333-µm mesh)	2.36 ± 0.37 (up); 5.73 ± 0.85 (down)	0.48 ± 0.09 - 5.92 ± 1.14 (up); 0.80 ± 0.30 - 11.22 ± 1.53 (down)	PE, PP, PS, ethylene	py-GCMS
Miller et al. (2017)	142	142	3 L (grab)	490	na	PET ~ PTFE >> PP ~ nitrocellu-lose/clay	µ-FTIR
Leslie et al. (2017)	3	3	4.60 – 24.96 m^3 (continuous centrifugation)	100 (Meuse); 500 (Rhine1); 300 (Rhine2)	na	Not reported	µ-FTIR
Dris et al. (2018)	1	12	2.21 m^3 [winter]; 2.72 m^3 [summer] (plankton net)	66.2 [W]; 30.4 [S]	38.2 - 101.6 [W]; 18.7 - 30.4 [S]	PET >> PP > PA ~ PET/PUR blend	FTIR
	1	15	2.1 m^3 [LB]; 3 m^3 [RB]; 5.4 m^3 [MU]; 4 m^3 [MM & MB] (plankton net)	na	16.8 - 24.7 [MU]; 19.0 - 20.2 [MM]; 13.7 - 19.1 [MB]; 52.4 - 87.7 [LB]; 32.1 - 46.4 [RB]		
	5	95	0.2 - 4.0 m^3 (plankton net)	100.6 [P1]; 48.5 [P2]; 27.9 [P3]; 27.9 [P4]; 22.1 [P5]	5.7 - 398.0 [P1]; 2.7 - 441.4 [P2]; 3.2 - 92.2 [P3]; 2.4 - 156.6 [P4]; 1.0 - 85.0 [P5]		
Kapp and Yeatman (2018)	28	29 (grab); 29 (net)	1.85 L (grab, glass jar); 3,207 L (plankton net 100-µm mesh)	910 ± 1.14x10^3 (grab); 2.57 ± 2.95 (net)	0 - 5,405 (grab); 0 - 14 (net)	PP, PE, PET, PEST	µ-Raman
Lahens et al. (2018)	6	7 (grab); 6 (net)	0.27-0.30 L (grab); 0.47-3.18 m^3 (300-µm mesh net, towed 60 s)	na	Fibers: 1.72x10^5 (BC) - 5.19x10^5 (KT). Fragments: 10 (BC) - 223 (LG)	Fibers: PES >>> PET > PE ~ PP ~ PE/PP ~ rayon. Fragments: PE > PP >PE/PP >> PS > PA ~ PVC	µ-FTIR
Lin et al. (2018)	14	14	60 L (bulk; 5-L vertical water sampler)	2,724	379 - 7,924	PP > PE ~ PET	µ-FTIR
Rodrigues et al. (2018)	3	12	1.2 m^3 (pump)	na	6 – 1,265	PE, PP > PS, PET	ATR-FTIR
Cheung et al. (2019)	1	3	143, 185, 215 m^3 (rectangular device, PVC frame, 0.27-mm mesh net)	7.428 ± 3.678	1.30 - 14.02	PP/EPR >>>> PP >> PE ~ LDPE	ATR - FTIR

DO NOT SHARE

Table S2. MNPs in rivers (cont'd)

Author (year)	Sampling sites (#)	Samples (#)	Sample volume (technique)	Concentration (MPs/m^3) Mean	Range	Polymer(s)	Polymer ID method
Mani et al. (2019)	9	18	87±17 m^3 (Manta trawl, 300 µm mesh)	na	0.03 - 9.2 (P1-9)	PS	µ-Raman or ATR-FTIR
Amrutha et al. (2020)	14	24	125 L (10-L S-S bucket)	288	56 - 2,328	PE >> PET >>> PP >>> PVC	FTIR
Grbic et al. (2020)	3	9	4 L (stainless steel bucket)	$1.54x10^4 \pm 7.90x10^3$	na	Tire/road wear > Cellulose ~ PE ~ Acrylic > PP ~ PVC additive > PET ~ PS ~ Nylon ~ Polyamide	ATR-FTIR and µ-Raman
Liu et al. (2020)	30	121	198.05 - 781.50 m^3 (Manta trawl) and 20 L (pump)	14.17 ± 14.64 (Manta trawl); $9.30x10^3 \pm 4.72x10^3$ (pump)	0.69-74.95 (Manta trawl); $2.64x10^3$ - $2.06x10^4$ (pump)	PE > PE-PP blend > Polyamide > PP > PS> > PU >> PET	µ-FTIR
Pan et al. (2020)	7	7	20 L (10-L bucket)	246	50 – 725	PP >> PE >>> PE-PP blend, PES, PS, PET	µ-Raman
Rowley et al. (2020)	2	69	N/A (ichthyoplankton net, 250 µm mesh)	24.8 (up-s); 14.2 (down-s)		PP, PE (low, medium, high densities)	ATR-FTIR
Scherer et al. (2020)	11	10	3.2 – 32.7 m^3	5.57 ± 4.33	0.88 – 13.24	PE ~ PP >>> PS	ATR-FTIR
Zhang et al. (2020)	20	40	10 L (pump)	2,345 ± 1,858	500 - 7,700	Fibers: PE ~ PET > nylon > PP. Fragments: PE > PP	Raman
Zhou et al. (2020)	7	21	25 L (steel sampler)	na	$9.12x10^2 \pm 2.00x10^2$ to $3.40x10^3 \pm 7.07x10^2$	PP > PE > PS >> PVC	FTIR
Bujaczek et al. (2021)	8	22	5.5 m^3 ave. (8" nylon plankton net 53 µm mesh)	26.2 ± 18.4	4.6 – 88.3	Fibers: PES >>> cellulose acetate ~ PP. Fragments: PE >>> PP >> PE/PP. Films: Polyvinyl acetate >>> PP	µ-Raman
Chen et al. (2021)	10	480 (R); 20 (T); 3 (24-h R compo-site); 5 (R depth)	10 L (2-L bottle, R and T); sampler, R composite)	$4.39x10^3 \pm 5.11x10^3$ (R); $4.59x10^4 \pm 2.48x10^4$ (T)	na	PET >> HDPE > LDPE > PP ~ EVA > PVDC ~ PS	FTIR

DO NOT SHARE

Table S2. MNPs in rivers (cont'd)

Author (year)	Sampling sites (#)	Samples (#)	Sample volume (technique)	Concentration (MPs/m^3) Mean	Range	Polymer(s)	Polymer ID method
He et al. (2021)	10	90	40 L (S-S bucket)	1,635	800.0 ± 300.0 to 3088.9 ± 330.6	PP > PE >> PS ~ PE/PP > PVC	ATR-FTIR
Mai et al. (2021)	8	24	5 L (S-S bucket)	na	$4.00x10^3$ ± $1.16x10^3$ to $1.53x10^4$ ± $6.70x10^2$	POE > PET > PUA > EP > PF >> PA ~ PE	µ-FTIR
Montecinos et al. (2021)	1	3	5 L (5-L PP bucket)	$8.7x10^6$	na	na	µ-Raman
Napper et al., (2021)	10	60		51 ± 7		Rayon >> acrylic >>> PET, PVC, PEST, nylon	ATR-FTIR
	10	60		26 ± 4	na		
Schell et al. (2021)	3	9	10,000 L (submersible pump)	na	1.3 - 227.0	PE >>> PP >>> tire wear (PES, Acrylic, PS in small amounts)	µ-FTIR
Sekudewicz et al. (2021)	3	3	20 L (S-S water probe)	na	$1.6x10^3$ - $2.6x10^3$	Fibers: PS ~ PP >>>> PE > nylon	Raman and/ or µ-FTIR
Wang et al. (2021)	13	78	2.5 L (S-S sampler)	Dry: $1.5x10^4$ ± $3.0x10^3$ (U); $1.7x10^4$ ± $7.0x10^3$ (M); $1.7x10^4$ ± $2.0x10^3$ (D). Wet: $1.3x10^4$ ± $2.0x10^3$ (U); $1x10^4$ ± $1x10^3$ (M); $1.4x10^4$ ± $3.0x10^3$ (D)	na	Dry: PP > PE ~ PS > PET > PVC ~ PA; Wet: PP ~ PET > PS ~ PE >PA ~ PVC	µ-FTIR
Zhang et al. (2021)	13	13	90 L (Teflon pump)	67.5 ± 65.6	~10 – 175	Flakes: PP/PE > PE > PP >> PB > PVC; Fibers: PP/PE > PP ~ PET > PA >> PB > PES ~ PE	µ-FTIR
			62.25 - 725.70 m^3 (net 75 µm mesh)	0.67 ± 0.41	0.13 – 1.50		
			62.25 - 725.70 m^3 (net 300 µm mesh)	0.15 ± 0.15	~0.1 – 0.5		

DO NOT SHARE

Table S3. MPs in lakes, reservoirs, and retention ponds

MPs in lakes and reservoirs, and retentions ponds								
					Concentration (MPs/m^3)			
Author (year)	Country	Sampling sites (n)	Samples (n)	Sample volume (method)	Mean	Range	Polymer	Polymer identification method
Wang et al. (2019)	China	9	9	20 L (pump)	na	1,400 ± 390 to 10,120 ± 4,090	PE >> PS, PET	FTIR
Grbic et al. (2020)	Canada	4	12	10 L (bucket)	800 ± 700	na	Cellulose >> PET > PE > PVC, PP	µ-Raman
Uurasjarvi et al. (2020)	Finland	6	12	6.2±1.8 L, 83±37 L, 468±75 L (pump-filtration)	1.8 ± 2.3, 12 ± 17, 155 ± 73	na	Fragments: PE, PP, PMMA, PVC, PET, and PS; fibers: PP, PET, PAN	µ-FTIR
		8	8	58±14 m^3 (manta trawl)	0.27 ± 0.18	0.037 - 0.66		
Bertoldi et al. (2021)	Brazil	7	7	35 m^3 (net)		11.9 ± 0.6 to 61.2 ± 6.1	PP > PE >> PTFE, PA, PU, PS	µ-FTIR and µ-Raman
Negrete Velasco et al. (2020)	Switzerland	2	2	5 L (5-L glass jar)	2,600; (MFs: 5,200 [H], 3,600 [O])	na	MPs: PE > PP >> PB, PBMA, PET, PS; MFs: PP, PS	ATR-µ-FTIR
Dong et al. (2021)	China	11	22	40 L (10-L bucket)		375 - 1,310	PET > PP > nylon > PAN, UF > PEP/PEPD > PVC, PE	µ-FTIR
Di et al. (2018)	China	29	58	25 L (pump)	4,703±2,816 (rural: 3,208±1,540; suburb: 4,366±2,855; urban: 6,201±3,034)	1,597 - 12,611	PP > PE > PS >> PVC, PC	µ-Raman
Di et al. (2019)	China	20	40	20 L (pump)	2,594 ± 3,875 (Res 1: 1,406 ± 764; Res 2: 1,457 ± 908; urban: 6,087 ± 6,981)	467 - 15,017	PP > PS > PE	µ-Raman
Tan et al. (2019)	China	4	12	31.4 m^3 (net)	0.56 ± 0.45	0.28±0.06 to 1.11±0.66	EPS > PE > PP > PS > PVC > PET	µ-FTIR
Lin et al. (2021)	China	13	39	5 L (S-S sampler)	7,205 (total); 6,081 (top); 9,777 (mid); 5,757 (bottom)	530 - 24,798 (all); 637 - 12,466 (top); 5,058 - 24,798 (mid); 530 - 15,720 (bottom)	PE > PA > PVF > PP > PVC > PS	Raman
Wang et al. (2021)	China	6	36 (18 per season)	2.5 L (S-S sampler)	na	Dry: $1.3x10^4$±$4.0x10^3$ (Res 2) - $2.2x10^4$±$5x10^3$ (Res 1). Wet: $1.3x10^4$± $1.0x10^3$ (Res 6) - $1.9x10^4$ ±$2.0x10^3$ (Res 1)	Dry: PET > PP, PS, PA > PE > PVC > PC; Wet: PET > PP > PE, PS, PA > PVC, PC	µ-FTIR
Olesen et al. (2019)	Denmark	1	5	10 L (5-L gb)	$2.6x10^5$ ($3.4x10^3$ µg/m^3)	na	PEST > acrylic, PP > PA > PE > PS (number); PP > PEST > acrylic > PA, PE, PS (mass)	µ-FTIR

Note. Glass bottle (gb), reservoir (Res), stainless-steel (S-S), not available (na)

DO NOT SHARE

Table S4. MPs in effluents of wastewater treatment plants

				Concentration (MPs/m³)			
Author (year)	Sampling sites (n)	Samples [effl.] (n)	Sample volume (method)	Mean	Range	Polymer	Polymer identification method
Murphy et al. (2016)	4	1	50 L (10-L S-S bucket) [effl.]	250 ± 40	na	PESTs > PA > PP, acrylic > alkyd > PET, PE, PS, PVA, polyaryl ether	µ-FTIR
Blair et al. (2019)	8	5	5 L	na	$<1x10^3$ - $3x10^3$	PP >> PVS > PE > POM	ATR-FTIR
Bayo et al. (2020)	4	36	4 L (gb) [effl.]	310 ± 60 [effl.]	na	LDPE > acrylate, HDPE, PET, PP > MCR, nylon, PES, PEP, PV > MUF, PS, PTFE	FTIR
Ding et al. (2020)	2	1	100 mL	$6.2x10^4$ (MPs, µ-beads); $7x10^3$ (µ-beads) [effl.]	na	Acrylic resin, PP (Exf srb); acrylic resin, PE (Exf thp)	FTIR
Edo et al. (2020)	2	3	25 L (25-L HDPE b) [effl.]	$1.28x10^4$ ± $6.30x10^3$ [effl.]	$<10^3$ - $2.84x10^4$ [effl.]	PE >> PESTs (PET, PP, cellophane)	µ-FTIR
Wang et al. (2020)	18	9	2 L	na	$6x10^3$ – $2.6x10^4$	PS, PE, PP >> PET > PA	µ-Raman
	5	5	2 L	na	$8x10^3$ – $4x10^4$	PE, PP, PS >> PET	
Grbic et al. (2020)	3	9	4 L (auto-sampler)	$1.33x10^4$ ± $1.55x10^4$	na	Cellulose >> PET > PE > PVC, PVA, PP, PU	µ-Raman & ATR-FTIR
Pittura et al. (2021)	4	1	25 L (auto-sampler)	520	na	PUR, EPM > PE, PESTs	µ-FTIR
	2	1	25 L (auto-sampler)	200	na	PESTs	
Prajapati et al. (2021)	1	6	50 L (Plankton net, 20-µm)	$1.69x10^3$ ± 70 (winter); $1.76x10^3$ ± 30 (spring); $1.84x10^3$ ± 230 (summer)	na	MPs: HDPE, MDPE > PE > PET, PMMA; MFs: PESTs	µ-FTIR (MPs) & µ-Raman (MFs)
Schell et al. (2021)	10	10	200 L [effl.]	na	45 - 535	PE > PP > PES > acrylic > PS	µ-FTIR

Note. Effluent (effl.), exfoliant scrub (Exf srb), exfoliant toothpaste (Exf thp), glass bottle (gb), HDPE bottle (HDPE b), stainless-steel (S-S), not available (na)

DO NOT SHARE
